# Multi-Omics Landscape of Circadian Clock Dysregulation Across the Chronic Liver Disease Spectrum

**DOI:** 10.3390/ijms27104571

**Published:** 2026-05-19

**Authors:** Sen Tong, Wenling Chen, Jiaxin Chen, Xiaosong Zhu, Anhua Shi

**Affiliations:** 1 Yunnan Key Laboratory of Integrated Traditional Chinese and Western Medicine for Chronic Disease in Prevention and Treatment, Yunnan University of Chinese Medicine, Kunming 650500, China; tongsen@ynucm.edu.cn (S.T.); ynwenling09@126.com (W.C.); chenjiaxin@ynucm.edu.cn (J.C.); 2 Key Laboratory of Microcosmic Syndrome Differentiation, Yunnan University of Chinese Medicine, Kunming 650500, China; 3 The First Clinical College, Yunnan University of Chinese Medicine, Kunming 650500, China

**Keywords:** circadian clocks, liver diseases, multi-omics, ARNTL transcription factors, epigenomics

## Abstract

The liver circadian clock coordinates hepatic lipid metabolism, bile acid synthesis, and glucose homeostasis through interlocking transcription–translation feedback loops. Disruption of this temporal organization is increasingly recognized as a shared pathological feature across the chronic liver disease spectrum. Transcriptomic profiling alone cannot capture the full scope of circadian dysregulation. Approximately half of rhythmically abundant hepatic proteins lack correspondingly rhythmic mRNAs. Roughly 25% of hepatic phosphosites oscillate with a 24-h period. Integrating transcriptomics, proteomics, post-translational modification profiling, metabolomics, and emerging single-cell and spatial approaches is therefore necessary for an accurate account of how circadian programs are remodeled in disease. This narrative review delineates the multi-omics landscape of circadian clock dysregulation across six chronic liver disease categories. These encompass metabolic dysfunction-associated fatty liver disease (MAFLD), alcoholic liver disease (ALD), viral hepatitis, hepatocellular carcinoma (HCC), liver fibrosis, and cholestatic disease. Four molecular features recur across these contexts. BMAL1 functional downregulation, REV-ERBα oscillatory output attenuation, NAD^+^ oscillatory amplitude reduction, and gut–liver axis circadian desynchronization together constitute an inferential framework for hepatic circadian failure. These features represent recurring disease-associated motifs rather than an established pan-disease mechanism. The upstream mechanisms and evidence depth differ substantially by disease category. Oncogenic kinase-driven CLOCK post-translational modifications in HCC, phosphoproteomic remodeling in MAFLD, and epigenomic clock disruption persisting after HCV clearance represent findings that transcriptomics alone would not resolve. The near-complete absence of temporally resolved human tissue data remains the principal barrier to translational progress. This evidence gap limits the clinical actionability of current mechanistic findings across all disease categories. Circadian phase inference algorithms and prospective temporally designed cohort studies offer a methodologically grounded path toward clinically actionable circadian hepatology.

## 1. Introduction

The liver is the principal metabolic organ of the vertebrate body. It coordinates lipid synthesis, carbohydrate storage, and bile acid production in response to nutritional demand. These functions follow predictable 24-h oscillations driven by the circadian clock [1]. At the molecular level, BMAL1 (Brain and muscle ARNT-like protein 1) and CLOCK (Circadian locomotor output cycles kaput) form the primary activator complex of the hepatic circadian clock [2]. This complex drives rhythmic expression of the repressors Period 1 and 2 (*PER1* and *PER2*) and Cryptochrome 1 and 2 (*CRY1* and *CRY2*). These repressor proteins then inhibit their own transcription, completing the primary oscillatory loop. A stabilizing auxiliary loop involves REV-ERBα (nuclear receptor subfamily 1 group D member 1, encoded by *NR1D1*) and RORα (RAR-related orphan receptor alpha, encoded by *RORA*). Together, REV-ERBα and RORα reinforce oscillatory amplitude and precision [3]. This machinery drives rhythmic transcription of approximately 10–20% of expressed hepatic protein-coding genes. It controls the temporal organization of de novo lipogenesis, bile acid synthesis, and glucose homeostasis [4,5]. Approximately 90% of rhythmically expressed hepatic transcripts depend on systemic entraining cues beyond those generated within the hepatocyte. Feeding–fasting cycles represent the dominant zeitgeber for hepatic circadian alignment [6,7].

When this temporal organization breaks down, the pathological consequences extend across the full chronic liver disease spectrum. Evidence consistently links circadian disruption to accelerated liver pathology across multiple disease stages [8,9]. Clock dysfunction has been documented in metabolic dysfunction-associated fatty liver disease (MAFLD), alcoholic liver disease (ALD), viral hepatitis, hepatocellular carcinoma (HCC), liver fibrosis, and cholestatic disease. Across these conditions, circadian failure operates through distinct but partially overlapping mechanisms. In MAFLD, BMAL1 disruption impairs lipid rhythmicity and accelerates steatosis, establishing a bidirectional cycle of metabolic deterioration [10,11]. In ALD, ethanol suppresses hepatic clock gene expression and uncouples gut microbiota rhythms from hepatic metabolic output [12,13]. Hepatitis C virus (HCV) infection perturbs the rhythmic transcriptome of human hepatocytes across more than one thousand genes [14]. HBV entry efficiency is gated by REV-ERBα-dependent rhythmic expression of the sodium taurocholate cotransporting polypeptide (NTCP) receptor [15]. In HCC, oncogenic kinase signaling redirects CLOCK protein toward cytoplasmic nucleotide synthesis, driving tumor growth independently of transcriptional clock output [16,17]. These findings collectively establish clock dysregulation as a shared pathological feature across the chronic liver disease spectrum.

Understanding this spectrum requires analytical approaches that match the complexity of circadian regulation. Transcriptomic profiling has been the dominant tool, yet it captures only a fraction of the regulatory landscape. Approximately half of all rhythmically abundant liver proteins lack a correspondingly rhythmic mRNA, as established in mouse liver proteomics studies [18]. This post-transcriptional decoupling indicates that transcript-level measurements substantially underestimate the functional circadian proteome. In mouse liver, approximately 25% of quantified phosphorylation sites oscillate with a 24-h period, and their rhythmic amplitude frequently exceeds that of corresponding mRNAs [19]. Rhythmic acetylation of mitochondrial proteins links NAD^+^ oscillations to metabolic enzyme activity through a regulatory layer invisible to transcriptomics alone [20]. Evidence also suggests that non-transcriptional oscillatory mechanisms may sustain circadian organization independently of the canonical feedback loop, though independent verification in disease-relevant liver models remains awaited [21]. Circadian profiles of more than 500 hepatic metabolites have been mapped in mouse liver, and some metabolite oscillations persist even when transcriptional output is substantially curtailed. Metabolomics should therefore be treated as an independent evidential layer rather than a downstream proxy for transcriptional activity. Single-cell RNA sequencing and spatial transcriptomics further reveal cell-type-specific and spatially resolved circadian programs that population-level measurements cannot detect [22]. No published study has yet applied these approaches to clock gene expression in any liver disease context, making them a high-priority direction for future investigation.

Prior reviews have catalogued associations between circadian disruption and liver pathology [23]. However, they have not engaged with the multi-omics evidence base in sufficient depth. Conversely, the most rigorous multi-omics circadian studies have been conducted in healthy animals under controlled conditions, without extension to disease states. This review addresses that gap by presenting multi-omics evidence organized by disease category. It covers MAFLD and steatohepatitis, viral hepatitis and HCC, and fibrosis together with ALD and cholestatic disease. This review differs from prior work in three respects. It integrates evidence from transcriptomics, proteomics, post-translational modification profiling, metabolomics, and emerging single-cell and spatial approaches within a disease-organized analytical framework. It proposes four molecular features that recur across disease categories as an inferential framework rather than an established pan-disease mechanism. These features are BMAL1 functional downregulation, REV-ERBα output attenuation, NAD^+^ amplitude reduction, and gut–liver axis desynchronization. It also explicitly maps where the evidence is mature and where critical gaps remain, with the aim of orienting future human-focused research. A translational discussion addresses circadian biomarker development and clock-targeted therapeutic directions, with attention to the current limits of human evidence. The literature was identified through PubMed and Web of Science searches, with no fixed date restriction, using terms related to circadian clocks, liver disease, and omics approaches. This is a narrative review. The depth of coverage across disease categories reflects the underlying evidence base, as available data differ substantially across disease types and omics layers.

## 2. The Multi-Omics Framework for Studying Hepatic Circadian Dysregulation

**Figure 1:**
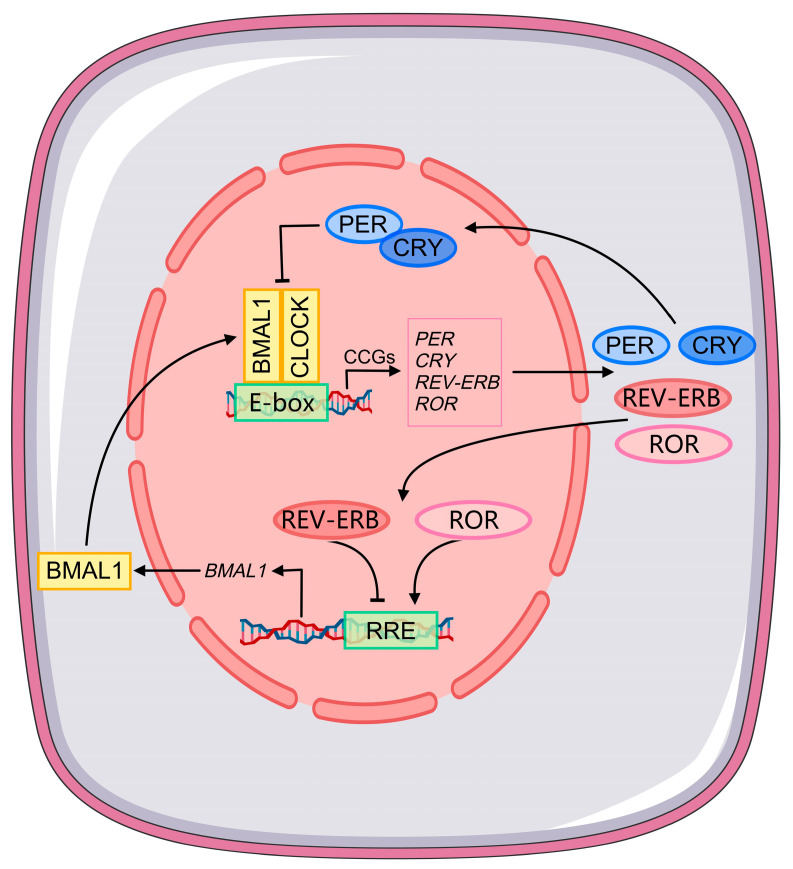
The interlocking transcription–translation feedback loops of the mammalian hepatic circadian clock. The primary loop operates through the BMAL1/CLOCK heterodimer, which binds E-box regulatory elements to drive rhythmic transcription of clock-controlled genes including PER, CRY, REV-ERB, and ROR. PER and CRY proteins accumulate in the cytoplasm, translocate to the nucleus, and inhibit BMAL1/CLOCK transcriptional activity, completing the approximately 24-h oscillatory cycle. The auxiliary stabilizing loop operates through nuclear REV-ERB and ROR proteins, which compete at RRE elements in the BMAL1 promoter to suppress or activate BMAL1 transcription, respectively, reinforcing oscillatory amplitude and periodicity. CCGs, clock-controlled genes; RRE, ROR response element.

Circadian regulation in the liver operates across at least three partially autonomous molecular layers. The transcriptome, the proteome with its post-translational modifications, and the metabolome each carry rhythmic information. They are not simply sequential outputs of a single upstream oscillator (Figure 1). Transcriptomic profiling across the circadian cycle has catalogued thousands of rhythmically expressed hepatic genes and established temporal maps of metabolic pathway activation. Yet the explanatory reach of the transcriptome is bounded. Approximately half of all rhythmically abundant liver proteins lack a correspondingly rhythmic mRNA [18]. This post-transcriptional decoupling indicates that post-transcriptional and post-translational mechanisms account for a large fraction of the functional circadian proteome.

Of more than 20,000 phosphorylation sites quantified in mouse liver, approximately 25% oscillate with a 24-h period [19]. Their rhythmic amplitude frequently exceeds that of corresponding mRNAs. These protein-level and phosphorylation-level rhythms can persist even when transcript oscillations are substantially attenuated. Transcriptomic assessment of disease states therefore carries a measurable risk of underestimating the true scope of circadian dysregulation. Rhythmic acetylation of mitochondrial proteins constitutes a distinct post-translational modification layer, linking NAD^+^ oscillations to metabolic enzyme activity through a channel that transcriptomics cannot detect [20]. The SIRT1 and SIRT3 deacetylases translate rhythmic NAD^+^ availability into coordinated adjustments of fatty acid oxidation, TCA cycle flux, and oxidative phosphorylation [24,25].

The question of whether circadian oscillations can persist independently of the canonical transcription–translation feedback loop has attracted considerable experimental attention. Ray and colleagues reported that circadian rhythms in the hepatic proteome and phosphoproteome may be detectable in *Bmal1*-null animals [21]. This observation was interpreted as evidence for non-transcriptional pacemakers that sustain oscillatory organization independently of the canonical loop. However, a published reanalysis contested the transcriptomic component of this finding on methodological grounds, specifically regarding fibroblast rather than hepatic data [26]. Whether non-transcriptional oscillators operate with functional significance in disease-relevant liver models therefore remains an open question. Independent experimental verification in hepatic tissue has not yet been reported. The mechanistic interpretation of existing data should therefore be regarded as a working hypothesis rather than an established feature of liver circadian biology.

**Figure 2:**
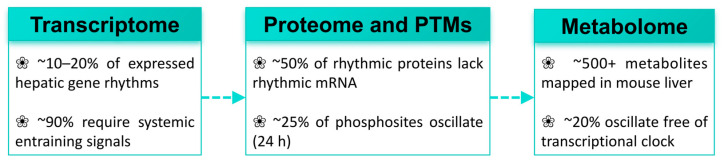
The three partially autonomous molecular layers of hepatic circadian regulation and their temporal relationships. The transcriptome, proteome with post-translational modifications, and metabolome each carry independent rhythmic information with temporal offsets relative to one another. Approximately 50% of rhythmically abundant liver proteins lack a correspondingly rhythmic mRNA, and some metabolite oscillations persist when transcriptional output is substantially curtailed. Dashed arrows indicate directional regulatory influence rather than complete dependency. Quantitative benchmarks are derived from published mouse liver studies.

Circadian profiles of more than 500 hepatic metabolites have been mapped in mouse liver [27]. Some metabolite oscillations are maintained even when the transcriptional program is substantially curtailed. This partial independence from transcriptional output positions metabolomics as a distinct evidential layer rather than a downstream proxy for clock gene activity. Each of the three molecular layers carries rhythmic information that the others cannot fully substitute. Their temporal offsets relative to one another have mechanistic consequences for how circadian dysregulation manifests in disease. The organizational relationships among these layers, including the key quantitative benchmarks and their directional dependencies, are summarized in Figure 2.

Single-cell RNA sequencing and spatial transcriptomics represent high-priority methodological directions for future investigation of hepatic circadian dysregulation. Bulk omics approaches average signals across hepatocytes, Kupffer cells, hepatic stellate cells, and sinusoidal endothelial cells. Each of these cell types carries a distinct circadian program that population-level measurements cannot resolve [22]. Single-cell profiling would allow direct characterization of cell-type-specific clock gene dysregulation in diseased tissue. It would reveal, for instance, the extent to which non-parenchymal cells lose or acquire rhythmic programs independently of the hepatocyte oscillator. The hepatocyte clock exerts paracrine temporal control over neighboring non-parenchymal cells, and disease-associated remodeling of that paracrine signaling may differ fundamentally by cell type. Spatial transcriptomics further reveals that many hepatic genes are simultaneously zonated and rhythmic, with multiplicative space–time effects that bulk sampling cannot detect [28]. In the context of liver disease, spatial profiling could address a specific structural question. It would clarify whether clock gene dysregulation follows the periportal-to-pericentral gradient of hepatic injury or reflects a more diffuse reorganization across the lobular architecture. To date, no published study has applied single-cell or spatial transcriptomic profiling to clock gene expression in any liver disease context. Integrating these approaches with bulk multi-omics data would substantially advance understanding of how circadian programs are remodeled across the liver disease spectrum.

## 3. Circadian Clock Dysregulation Across the Liver Disease Spectrum

Chronic liver disease encompasses a spectrum of distinct etiologies, yet circadian clock dysregulation emerges as a shared pathological axis across metabolic, viral, fibrotic, and cholestatic conditions. At the level of clock gene output, a recurring pattern is identifiable. BMAL1 transcriptional activity is functionally attenuated, REV-ERBα oscillatory output is dampened, and CLOCK:BMAL1-driven target gene rhythmicity is disrupted by inflammatory interference. These features recur across disease contexts and represent the basis for the inferential framework proposed in this review. However, the depth of supporting evidence differs substantially across disease categories. Transcriptomic and epigenomic data are comparatively mature for MAFLD and HCV hepatitis, while time-resolved proteomic and metabolomic data remain absent for HBV-related disease and cholestatic liver disease. The discussions that follow identify the evidence source and its limitations for each major conclusion. The predominant clock gene alterations identified across MAFLD, viral hepatitis, HCC, liver fibrosis, ALD, and cholestatic disease are summarized in Table 1. The multi-omics evidence underlying these alterations is presented across three disease groupings, addressing MAFLD and steatohepatitis, viral hepatitis and HCC, and fibrosis together with ALD and cholestatic disease.

### 3.1. Circadian Clock Dysregulation in Metabolic-Associated Liver Disease

#### 3.1.1. Transcriptomic and Proteomic Remodeling in Hepatic Steatosis and Steatohepatitis

The transcriptomic consequences of BMAL1 loss in hepatocytes have been characterized in liver-specific knockout models. In these animal studies, BMAL1 functions as an obligate organizer of hepatic lipid synthesis and fatty acid oxidation programs. In *Bmal1* liver-specific knockout mice, rhythmic transcription of *SREBP1c* target genes governing de novo lipogenesis is profoundly disrupted. PPARα-driven fatty acid oxidation programs are simultaneously suppressed, uncoupling anabolic and catabolic lipid pathways from their temporal coordination. Valcin and colleagues demonstrated in this mouse model that diurnal rhythms in *Srebf1*, *Ppara*, and downstream effectors including *Fasn*, *Scd1*, *Cpt1a*, and *Acox1* are all attenuated following hepatocyte-specific *Bmal1* deletion [40]. Zhang and colleagues confirmed that synthetic PPARα ligands partially restored fatty acid oxidation in *Bmal1*-deficient mouse liver [41]. The severity of this phenotype is most pronounced under high-fat dietary or ethanol challenge conditions in animal models. Direct human liver data confirming equivalent transcriptomic disruption under comparable conditions are not yet available.

Metabolic stress reciprocally attenuates circadian clock gene expression, establishing a bidirectional relationship between clock dysfunction and hepatic pathology. In mouse models, a high-fat diet reorganizes CLOCK:BMAL1 chromatin recruitment, impairing canonical clock output while activating surrogate PPARγ-driven oscillatory pathways. In genetically obese *db*/*db* mice, the amplitude of hepatic PER2 oscillations is dramatically reduced. Treatment with a ROR agonist restored PER2 amplitude and simultaneously reduced *Srebp1c* and inflammatory cytokine expression in that animal model [42]. Human MAFLD tissue provides convergent transcriptomic evidence from a distinct and more clinically relevant evidence layer. In human liver biopsies, clock repressor genes including *PER2*, *PER3*, and *CRY2* are progressively reduced across the histological spectrum from steatosis to MASH. *NR1D2* and *RORA* expression amplitudes decline with advancing fibrosis stage in human tissue data. The relationship between BMAL1 expression and fibrosis severity requires careful interpretation because animal model and human tissue findings point in divergent directions. In certain mouse models, hepatocyte-specific *Bmal1* deletion confers relative protection from fibrosis, yet this finding is not replicated consistently across whole-body knockout and diet-induced models. In human MAFLD tissue, by contrast, reduced BMAL1 expression correlates with increased fibrosis severity. These divergent observations most plausibly reflect differences in model design, including knockout specificity and hormonal context, rather than a fundamental biological divergence between species. This model-dependent variability is an important interpretive limitation when applying animal model clock-fibrosis findings to human disease.

The inflammatory amplification of clock gene suppression operates through molecular pathways established primarily in cell-based and animal studies. In these experimental systems, TNF-α and IL-1β suppress *Per1*, *Per2*, *Dbp*, *Tef*, and *Hlf* transcription by directly impairing CLOCK:BMAL1-induced E-box activation. NF-κB activation further relocalizes CLOCK and BMAL1 occupancy toward NF-κB-enriched genomic sites. This diverts transcriptional activity away from clock-controlled metabolic gene networks [43]. The structural basis for this interference involves RELA binding to the transactivation domain of BMAL1. RELA competes with the coactivator CBP/p300, shortening circadian period and dampening oscillatory amplitude without abolishing the clock entirely [44]. BMAL1 normally restrains NF-κB activity through its transcriptional target REV-ERBα [45]. BMAL1 loss therefore removes a key anti-inflammatory brake, allowing NF-κB to sustain its own activating signal. NF-κB additionally drives DNMT1-mediated promoter methylation of *BMAL1*, potentially completing a self-amplifying pathological cycle. This epigenetic reinforcement mechanism is supported by indirect mechanistic evidence and has not yet been directly demonstrated in human MAFLD liver tissue.

**Figure 3:**
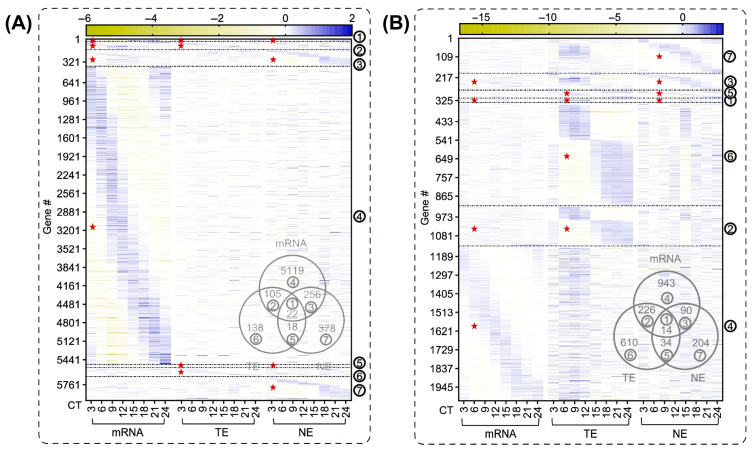
Post-transcriptional and post-translational mechanisms dominate hepatic circadian proteome organization under standard and environmentally disrupted conditions. Heatmaps of rhythmically expressed transcripts (mRNA), whole-cell proteins (TE), and nuclear proteins (NE) in mouse liver under standard conditions (**A**) and environmental circadian disruption (**B**). Only 22 genes display rhythmicity at all three molecular levels simultaneously under standard conditions. Environmental disruption rewrites rhythmicity across approximately 98% of the whole-cell proteome and 95% of the nuclear proteome. TE, total extract; NE, nuclear extract; Red star, rhythmic portion. Adapted from Duong et al. (2024) [46], Nature Communications, under CC BY 4.0.

The proteomic dimension of circadian remodeling in hepatic steatosis extends beyond what transcriptomic profiling can resolve. The quantitative benchmarks underlying this argument were established in healthy mouse liver and have not yet been directly replicated in disease states through time-resolved studies. Duong and colleagues demonstrated that severe experimental circadian disruption rewrote the rhythmicity of approximately 98% of the whole-cell liver proteome and 95% of the nuclear proteome in a mouse model [46]. That finding was obtained under extreme light-cycle perturbation conditions that differ substantially from the metabolic and behavioral context of clinical MAFLD. The magnitude of proteomic remodeling in human MAFLD is unlikely to reach equivalent levels. Nevertheless, these data indicate that the scope of circadian remodeling in metabolic liver disease has been substantially underestimated by transcriptomic studies alone (Figure 3).

Clock protein phosphorylation is directly coupled to the metabolic state of the hepatocyte through the NAD^+^ oscillatory system. SIRT1 deacetylates both BMAL1 and PER2 in a NAD^+^-dependent manner, linking rhythmic NAD^+^ availability to the stability and activity of clock proteins. NAD^+^ levels are depleted in MAFLD and MASH, and this depletion impairs SIRT1-mediated clock protein deacetylation. The consequence is aberrant PER2 accumulation and disrupted negative feedback within the primary oscillatory loop. Additionally, mTORC1 activation promotes BMAL1 phosphorylation through S6K1-dependent signaling [47]. mTOR hyperactivation in metabolic liver disease therefore alters BMAL1 phosphorylation stoichiometry in ways not captured by transcript-level measurements. The only published human liver tissue phosphoproteomic study in MAFLD was conducted by Wattacheril and colleagues, who profiled hepatic phosphorylation in biopsy-proven MAFLD patients across three histological groups [48]. They identified 53 phosphorylation sites altered more than twofold between groups. Enriched pathways distinguishing MASH from simple steatosis included carbohydrate metabolism and post-transcriptional RNA modification, both of which overlap with circadian-regulated phosphorylation networks identified in separate animal studies. That study represents the only available human liver phosphoproteomic dataset in MAFLD, and it was not designed with temporal resolution.

#### 3.1.2. Metabolomic Signatures of Disrupted Lipid, Bile Acid, and Amino Acid Rhythms

The hepatic metabolome oscillates with a 24-h periodicity that is partially independent of the transcriptional clock. This independence makes metabolite rhythm loss a distinct evidential layer in MAFLD, not simply a downstream echo of clock gene suppression. The quantitative basis for this argument was established in animal studies. Adamovich and colleagues used shotgun lipidomics across 159 lipid species in mouse liver [49]. Approximately 17% of lipid species oscillated circadianly. Triglyceride species displayed roughly twofold amplitude and peaked near CT8. In *Per1* and *Per2* double-knockout animals, an equivalent fraction of lipid species continued to oscillate. Their phases were completely inverted, with triglyceride peaks shifting to CT20. This inversion demonstrates that lipid metabolomic rhythms are sustained by feeding–fasting cycles independently of the canonical clock in mouse liver. Approximately 20% of hepatic metabolite oscillations were maintained in that model, compared with only 10% of transcript oscillations. Lipid metabolite rhythms specifically required inter-organ coordination for full restoration in these animal experiments. These findings suggest that hepatic lipidomics may reflect systemic circadian coherence rather than hepatocyte-autonomous transcriptional output.

Time-restricted feeding provides the most direct interventional evidence that lipid metabolomic rhythms are rescuable under metabolic disease conditions in animal models. Adamovich and colleagues showed that nighttime-restricted feeding increased the number of oscillating triglyceride species and reduced total hepatic triglyceride content by approximately 50% in mice [49]. Chaix and colleagues demonstrated that an 8-h feeding window reduced hepatic lipid accumulation even in *Cry1* and *Cry2* double-knockout and liver-specific *Bmal1*-knockout mice [50]. Mehus and colleagues documented that 12-h dark-phase restricted feeding on high-fat diet created a distinct lipid metabolic state with unique triglyceride and phospholipid species signatures in mouse liver [51]. Huang and colleagues identified clock regulation of fatty acid metabolism as the key diurnal feature reset by meal timing across six simultaneous omics layers in an animal study [52]. Together these interventional findings establish, in animal models, that the lipid metabolomic phenotype of circadian disruption is reversible through feeding schedule normalization independently of the molecular clock.

The bile acid dimension is organized around a mechanistic chain connecting clock output to enzyme rhythmicity and then to metabolite pool remodeling. CYP7A1 is the rate-limiting enzyme of the classical bile acid synthesis pathway. Its expression follows a robust circadian rhythm under the control of REV-ERBα. The clock output transcription factor D-box binding PAR bZIP transcription factor (DBP) independently transactivates both *CYP7A1* and the bile acid uptake transporter *NTCP*. RORα regulates *CYP8B1*, which determines the ratio of 12-hydroxylated to non-12-hydroxylated bile acids within the pool. RORα overexpression increases cholic acid and taurocholic acid concentrations in bile. MAFLD reduces both REV-ERBα amplitude and RORα activity in animal models. The 12-hydroxylated fraction of the bile acid pool has therefore been proposed as a metabolomic indicator of clock dysfunction at the enzyme level.

Ferrell and Chiang directly measured the metabolomic consequences of *CYP7A1* de-rhythmization in sleep-disrupted mice [53]. Peak *CYP7A1* expression was suppressed and phase-advanced by approximately four hours. The resulting bile acid redistribution was substantial in that animal model. Hepatic bile acids decreased during daytime, intestinal bile acids nearly doubled at ZT2, and gallbladder bile acids increased fivefold at ZT2. Eggink and colleagues showed that a high-fat diet combined with inactive-phase feeding abolished *CYP7A1* and *CYP8B1* expression rhythms entirely in rats [54]. Kettner and colleagues demonstrated that FXR ablation dramatically increased enterohepatic bile acid levels and accelerated hepatic steatosis induced by jet-lag protocols [55]. That study was conducted in a hepatocarcinogenesis model rather than a MAFLD model. Its findings should be understood as mechanistic context rather than direct evidence for bile acid clock disruption in metabolic liver disease. Within that interpretive constraint, the data position FXR de-rhythmization as a candidate mechanism amplifying circadian metabolomic disturbance into pathological bile acid accumulation.

The amino acid metabolome offers a mechanistically plausible but evidentially incomplete dimension of circadian disruption in MAFLD. Amino acid pathways were among the most enriched oscillating categories in that dataset. Branched-chain amino acids are elevated in MAFLD as a replicated metabolomic finding. Their accumulation activates mTOR signaling, which in turn alters BMAL1 phosphorylation stoichiometry. However, whether the diurnal oscillatory pattern of branched-chain amino acids is specifically disrupted in steatotic liver has not been directly measured. The proposed connection to clock dysfunction currently rests on indirect mechanistic inference rather than time-resolved metabolomic evidence. The one-carbon and transsulfuration pathways offer a more mechanistically grounded intersection of circadian regulation and amino acid metabolism. Cal-Kayitmazbatir and colleagues identified a direct physical interaction between CBS and CRY1 in mouse liver extracts [56]. This interaction provides molecular-level experimental evidence for bidirectional coupling between the clock and sulfur amino acid catabolism. Liver extracts from *Cry1*-null mice show reduced CBS enzymatic activity, and CRY1 reciprocally modulates CBS function through this physical association.

These findings suggest a plausible but evidentially incomplete framework in which MAFLD-associated clock disruption alters the temporal organization of sulfur amino acid catabolism and one-carbon metabolism. Direct time-resolved metabolomic confirmation in steatotic liver has not yet been reported in animal models or human cohorts. Across the three metabolite domains reviewed here, the evidence base differs substantially. Lipid and bile acid metabolomic rhythms are supported by multiple independent animal studies with consistent mechanistic findings. Amino acid metabolomic disruption remains at the level of mechanistic inference and requires direct experimental validation in disease-relevant models before equivalent conclusions can be drawn. Despite this heterogeneity, the available evidence points toward a shared principle. Metabolite oscillation loss in MAFLD reflects the combined failure of clock-dependent enzyme rhythmicity, impaired inter-organ circadian coordination, and disrupted feeding–fasting entrainment. Each of these three mechanisms operates as a partially independent contributor to the overall metabolomic phenotype.

#### 3.1.3. Gut Microbiota Circadian Oscillation and Its Hepatic Metabolic Consequences

The evidence reviewed in this section derives almost entirely from animal models and preclinical studies. The causal link between microbiota rhythm disruption and liver disease progression has not been established in human studies, and findings should be interpreted within that constraint. The gut microbiota exhibits diurnal rhythms in both community composition and metabolic output. These oscillations are maintained by host feeding–fasting cycles and intestinal clock gene activity. Thaiss and colleagues demonstrated in mice that fecal microbiota undergo consistent 24-h compositional fluctuations driven by host feeding behavior [57]. Ablation of host clock genes or experimental jet-lag induction disrupted these oscillations in that animal model. The resulting arrhythmic dysbiosis, when transplanted into germ-free recipients, was associated with obesity and glucose intolerance. This association suggests that disrupted microbiota rhythmicity may act as a potential mediator of metabolic dysfunction rather than a direct causal driver. A subsequent study from the same group showed that disrupted microbiota rhythmicity reprogrammed hepatic transcription on a genome-wide scale in mice [58]. Heddes and colleagues demonstrated equivalent effects using intestinal epithelial cell-specific Bmal1 ablation. Microbial oscillations persisted in constant darkness in that animal model, confirming true circadian rather than solely light-driven regulation [59].

Three classes of microbiota-derived signals hold the most direct mechanistic evidence for hepatic clock entrainment in preclinical models. Their molecular targets, mechanistic basis, and current evidence levels are presented in Table 2. The first class is short-chain fatty acids. Rhythmic SCFA production peaks approximately four hours after light onset and oscillates in phase with free fatty acid receptor expression in the colonic tissue of mice [60]. In *Bmal1*-knockout mice, both SCFA rhythms and receptor expression rhythms are abolished in that animal model. A related but chemically distinct signal, beta-hydroxybutyrate (BHB), is a ketone body originating from hepatic fatty acid oxidation. Tognini and colleagues demonstrated in mice that BHB drives cyclic histone deacetylase inhibition in the liver, altering BMAL1 chromatin recruitment in a feeding-phase-dependent manner [61]. That finding identifies ketone-mediated epigenetic signaling as a candidate mechanism influencing hepatic clock accessibility in animal models. Tahara and colleagues showed in mice that oral SCFA administration produced phase shifts in peripheral clocks including the liver [62]. Whether SCFA-mediated clock entrainment operates equivalently in human liver has not been directly tested in hepatic tissue. The second class of signals is secondary bile acids generated by microbial 7-alpha-dehydroxylation. Yao and colleagues used isogenic BSH-deleted and wild-type Bacteroides thetaiotaomicron in gnotobiotic animals. A single microbial enzymatic modification of bile acids substantially altered circadian pathway transcription in both gut and liver in that experimental system [63]. BSH activity itself oscillates in mouse gut microbiota with a feeding-entrained rhythm. The resulting temporal variation in secondary bile acid pool composition may serve as a potential time-of-day signal relayed to hepatic receptors including TGR5. This proposed relay mechanism awaits direct verification in human hepatic tissue [64]. The third class involves tryptophan-derived metabolites produced by intestinal bacteria. The aryl hydrocarbon receptor (AhR) provides the primary molecular interface between indole metabolites and the hepatic circadian machinery in cell-based and mouse studies. The AhR and its dimerization partner ARNT (aryl hydrocarbon receptor nuclear translocator) share the same PAS dimerization interface as the CLOCK-BMAL1 complex, creating structural competition at E-box regulatory elements [65,66]. Whether this AhR-mediated competition with BMAL1 operates with functional significance in human liver has not been directly demonstrated, and current evidence remains preclinical.

In MAFLD, the disruption of these three rhythmic communication channels may contribute to a self-amplifying cycle of gut–liver desynchronization. The mechanistic evidence for this cycle derives almost entirely from animal models. A high-fat diet abolished the diurnal rhythm of the host antimicrobial peptide *Reg3γ* in intestinal tissue of mice, impairing microbiota oscillatory dynamics and reducing rhythmic metabolite delivery to the liver [69]. Disrupted light–dark cycles and a high-fat diet together worsen both microbial dysrhythmia and hepatic steatohepatitis to a degree exceeding either insult alone in animal studies. One animal study reported that restoration of normal light–dark cycles after prolonged disruption paradoxically worsened hepatic steatosis and *Bmal1* expression in mice [70]. That finding comes from a single study with limited sample size and without temporal microbiota profiling. It should be regarded as a hypothesis-generating observation rather than an established mechanistic feature, and independent replication is needed before broader inferences can be drawn. Yang and colleagues provide the only available directional human evidence in this field. They documented disrupted plasma bile acid profiles and FGF19 rhythms in night-shift workers compared with day-shift controls through a cross-sectional plasma metabolomic study [71]. That study is currently the only human metabolomic dataset addressing gut–liver circadian axis disruption in a population with metabolic risk. It provides directional support for the animal model findings but cannot establish temporal causality or tissue-level clock-microbiota coupling due to its cross-sectional design.

### 3.2. Circadian Reprogramming in Viral Hepatitis and Its Progression to Hepatocellular Carcinoma

#### 3.2.1. Epigenomic and Transcriptomic Clock Remodeling in HCV Infection

**Figure 4:**
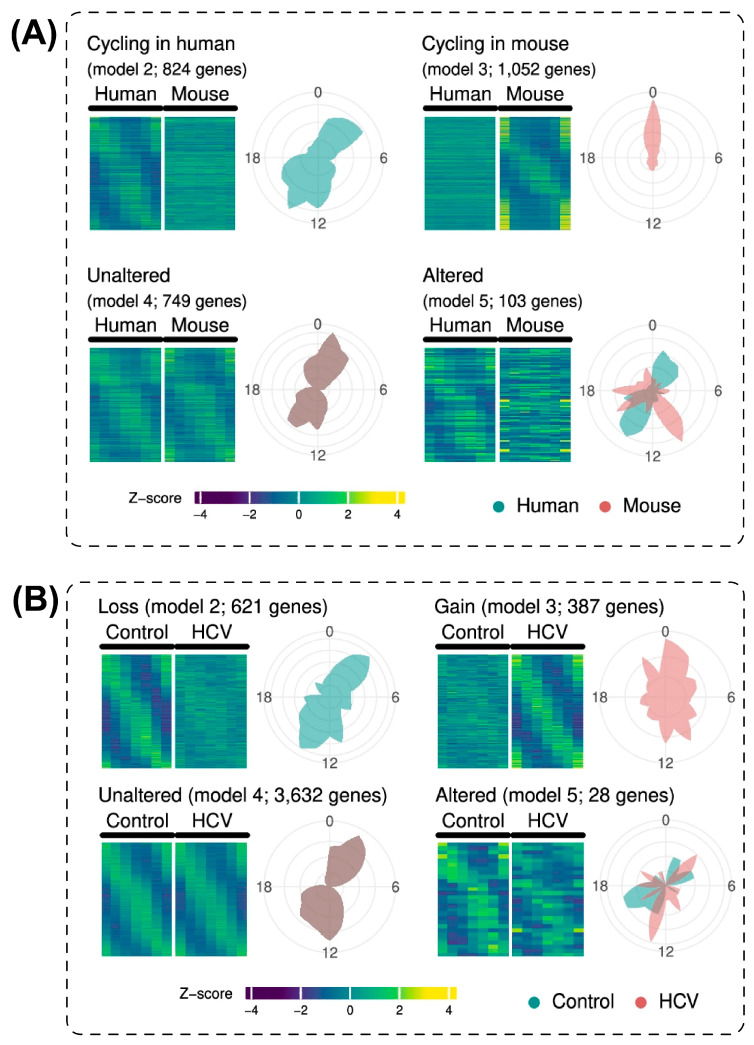
HCV infection perturbs the diurnal transcriptome of human hepatocytes across multiple oscillatory categories. Heatmaps and rose plots classifying rhythmically expressed genes in human liver chimeric mice by oscillatory category, comparing uninfected controls and HCV-infected conditions (**A**,**B**). HCV infection alters the rhythmicity of more than 1000 genes, encompassing loss of rhythmicity, de novo gain of rhythmicity, and alterations in phase or amplitude. HLCM, human liver chimeric mouse. Reproduced from Mukherji et al. (2024) [14], Nature Communications, under CC BY 4.0.

HCV infection remodels the temporal organization of the hepatic transcriptome across multiple functional gene categories. Mukherji and colleagues characterized this remodeling using a human liver chimeric mouse model [14]. In that system, immunodeficient mice were transplanted with primary human hepatocytes and infected with HCV genotype 1b. This model provides human hepatocyte-level data within an in vivo experimental environment. Its translational relevance exceeds that of standard rodent studies. However, findings from this model should not be equated with data derived directly from human liver tissue. The immunodeficient host environment and the absence of intact human immune–hepatic interactions represent important interpretive constraints. Animals were sampled at six timepoints across the 24-h diurnal cycle. RNA-seq analysis identified oscillatory alterations in more than 1000 protein-coding genes, representing approximately 22% of all rhythmically expressed transcripts in human hepatocytes. The perturbation encompassed loss of oscillation, de novo gain of rhythmicity, and alterations in phase and amplitude (Figure 4). Lipid metabolism gene networks lost their diurnal oscillatory organization. Fibrogenesis-related pathways including TGF-β and SMAD signaling gained aberrant rhythmic activity. A clock disruption signature derived from these chimeric model data was subsequently validated in a cohort of 216 patients with early-stage HCV cirrhosis, where it predicted overall survival. That human cohort validation represents a clinically direct evidence layer that is distinct from the chimeric model findings from which the signature was derived.

A bidirectional relationship between clock function and HCV propagation underlies this transcriptomic remodeling. BMAL1 drives rhythmic expression of HCV entry receptors including CD81 and claudin-1. Pharmacological REV-ERBα activation suppresses HCV RNA replication by reducing stearoyl-CoA desaturase activity [72]. That finding was established in cell-based and animal experiments rather than human clinical studies. In the reverse direction, HCV core protein downregulates *PER2* and *CRY2* mRNA levels and suppresses their corresponding proteins. This suppressive effect has been characterized for HCV genotype 1b and should not be assumed to apply across other genotypes. PER2 overexpression reduced HCV RNA replication by approximately 30% in that experimental system. It also partially restored interferon-stimulated gene expression patterns. HCV therefore suppresses PER2 to remove a constitutive antiviral constraint within the circadian machinery of the infected cell. A microRNA-mediated pathway reinforces this clock attenuation. Elevated miR-10a in HCV-infected hepatocytes suppresses RORα expression. This in turn attenuates *BMAL1* transcription and blunts its oscillatory amplitude [31]. That finding was established in human liver biopsy samples from HCV-related cirrhosis patients and represents direct human tissue evidence. Reduced *BMAL1* expression correlates with fibrosis stage in those biopsies and predicts HCC recurrence in HCV-related cirrhosis.

HCV-induced epigenomic remodeling extends beyond transcriptional output and shows evidence of persistence after viral clearance. Time-resolved ChIP-seq analysis in the chimeric liver model revealed perturbed rhythmic histone acetylation at clock-regulated metabolic gene loci [14]. These epigenetic marks were subsequently detected as stable modifications in clinical samples from patients who achieved sustained virological response with direct-acting antivirals [73,74]. That observation provides human-level evidence for post-clearance epigenomic persistence. HCV infection also elevates DNMT1 and DNMT3B activity in hepatocytes, as established in cell-based studies [75]. This provides a potential biochemical basis for promoter hypermethylation at clock-associated gene loci. However, direct methylation at those specific loci has not been demonstrated in the cited studies. Whether clock gene promoter CpG islands are directly targeted by this mechanism therefore remains unresolved. HCV infection induced DNA methylation changes across approximately 237 genes in the chimeric mouse model. That remodeling required the in vivo innate immune environment and was not reproducible under cell culture conditions. The available evidence reflects viral co-option of host regulatory intermediaries rather than direct disruption of clock protein function. Whether these epigenomic changes drive long-term hepatic disease risk after viral clearance requires further investigation in longitudinal human cohorts.

#### 3.2.2. Multi-Omics Landscape of Clock-Associated Gene Dysregulation in HCC

##### Transcriptional Dysregulation of Clock Genes in HCC

Transcriptomic profiling across independent HCC cohorts has established a consistent clock gene alteration pattern. *BMAL1*, *PER1*, *PER2*, *CRY2*, and *RORA* are downregulated in paired tumor and adjacent non-tumor tissue. *TIMELESS* and *NPAS2* are the most consistently upregulated components [76]. Promoter hypermethylation silences activator-arm clock genes across multiple cohorts [77]. miR-494-3p provides an additional post-transcriptional route for *BMAL1* suppression. The role of EZH2 in this silencing process remains directionally unresolved and should not be treated as an established mechanism [78]. In HBV-related HCC, HBx protein simultaneously upregulates positive-arm and suppresses negative-arm clock genes. That finding derives from a small-cohort transcript-level study and has not been independently replicated.

A critical interpretive constraint applies to all transcriptomic clock gene data in HCC. Reduced mean expression of a clock gene and loss of its oscillatory function are not equivalent phenomena. Yang and colleagues used XR-Seq profiling in DEN-induced mouse liver tumors and found that core clock gene rhythms are largely preserved despite altered expression levels [79]. Tumor-specific transcripts showed inverted phase relationships and MYC target enrichment. A tumor may therefore retain measurable rhythmic output while displaying downregulated mean clock gene expression.

##### Post-Translational Remodeling of Clock Proteins in HCC

The most consequential proteomic finding in HCC clock biology operates outside transcriptional regulation. Liu and colleagues demonstrated that IGF1R-driven CK2-mediated phosphorylation of CLOCK at serine 106 disrupts the nuclear CLOCK-BMAL1 complex in HCC cell lines [17]. Cytoplasmic CLOCK then acetylates PRPS1 and PRPS2, sustaining de novo nucleotide synthesis. CLOCK S106 phosphorylation and PRPS1/2 K29 acetylation associate with poor prognosis in human HCC specimens [80]. That prognostic association in human tissue represents a more clinically direct finding than the cell line mechanistic data. CRY2 downregulation in HCC destabilizes a parallel proteasomal checkpoint. CRY2 normally functions as a cofactor for the FBXL3-SCF E3 ubiquitin ligase complex, which recruits T58-phosphorylated c-MYC for degradation [81,82]. CRY2 loss therefore allows c-MYC to accumulate, and this mechanism is entirely post-translational.

Clock gene loss also disrupts cell cycle checkpoint control through protein-level mechanisms. PER2-dependent modulation of Wee1 kinase restrains Cyclin B1-CDC2 activity at the G2/M transition in carcinogen-exposed hepatocytes [83]. PER2 loss removes this clock-encoded mitotic gate in animal models. NPAS2 engages the same checkpoint from the opposing direction by transcriptionally activating *CDC25A* phosphatase in HCC cell lines [84]. No global phosphoproteomic, acetylome, or ubiquitome study has yet been conducted in HCC tissue.

##### Metabolic Reprogramming Through Clock-Associated Pathways in HCC

Clock-associated post-translational modifications extend into metabolic and oncogenic signaling networks. *FBXW7* disruption destabilizes REV-ERBα protein and perturbs hepatic lipid and glucose metabolic rhythms in experimental models [85]. *CSNK1D*, elevated in HCC tissue, activates Wnt signaling through DVL3 stabilization and contributes to sorafenib resistance in cell line studies [86]. Both findings derive from experimental systems rather than time-resolved human HCC tissue data.

Single-cell and spatial transcriptomic analyses have begun to resolve the cell-type distribution of clock gene dysregulation in HCC. Dysregulated circadian genes are predominantly distributed across malignant hepatocytes, cancer-associated fibroblasts, and tumor endothelial cells [87,88]. A pan-cancer single-cell analysis identified a 101-gene endothelial clock signature with independent prognostic value in HCC datasets [89]. Spatial profiling confirmed that clock-associated gene expression varies across HCC tissue architecture, with RBM17 elevated in tumor leading-edge regions [90]. These datasets were collected without circadian sampling design and therefore describe expression level distribution rather than oscillatory status. Time-resolved single-cell profiling in etiology-stratified HCC cohorts would be required to characterize cell-type-specific rhythmic disruption directly. The multi-omics evidence base for HBV-related HCC remains limited to small-cohort transcript-level findings across all three analytical layers discussed here. No time-resolved proteomic or metabolomic dataset exists for HBV-related HCC at any disease stage.

#### 3.2.3. Circadian Regulation of the HCC Immune Microenvironment

The relationship between circadian gene dysregulation and the HCC immune microenvironment is currently supported only by correlational and cross-cancer evidence. Zhang and colleagues applied TIMER-based immune deconvolution to TCGA HCC cohorts and found that *PER1*, *CRY2*, and *NPAS2* expression correlated with CD8+ T cell, dendritic cell, and macrophage infiltration patterns [91]. These correlations persisted after adjustment for tumor purity. However, correlation-based immune deconvolution cannot establish the direction or mechanism of any relationship between clock genes and immune cell recruitment. Those associations have not been validated in HCC-specific experimental models. The following evidence derives from non-hepatic tumor systems and should be understood as biological context rather than direct support for HCC-specific conclusions. Wang and colleagues demonstrated clock-dependent rhythmic CD8+ T cell infiltration and cytolytic function in murine and human tumors [92]. Fortin and colleagues identified rhythmic PD-L1 expression on MDSCs as a mechanism linking tumor circadian programs to CD8+ T cell suppression [93]. Whether equivalent clock-immune coupling operates within the HCC tumor microenvironment has not been directly demonstrated.

The proposition that clock gene downregulation promotes M2 macrophage polarization and immune evasion in HCC is best regarded as a hypothesis. It rests on two inferential steps across distinct biological contexts. The first draws on jet-lag experiments in a melanoma model, where circadian disruption abolished diurnal M1-to-M2 macrophage oscillations and accelerated tumor growth [94]. The second draws on BMAL1-macrophage polarization mechanisms characterized in ALD and immune-mediated hepatitis models. Extrapolating across both steps to the HCC tumor microenvironment involves compounding uncertainty that dedicated experimental investigation would need to resolve. The evidentiary gap in HBV-related HCC is particularly consequential.

### 3.3. Circadian Clock Dysregulation in Liver Fibrosis, ALD, and Cholestatic Disease

#### 3.3.1. Clock-Dependent Gating of Profibrotic Signaling and Hepatic Stellate Cell Activation

The hepatic circadian oscillator imposes temporal constraints on profibrotic signaling output, and the removal of these constraints is a defining feature of fibrosis-associated clock disruption. BMAL1 loss promotes HSC activation through two mechanistically independent pathways in cell-based and organoid models. The first operates through the IDH1 and α-ketoglutarate metabolic axis, through which BMAL1 suppresses aerobic glycolysis and blocks HSC proliferation and phenotypic transformation [95]. The second pathway operates through CK1ε-mediated phosphorylation of REV-ERBα and the cytoskeletal protein Transgelin in activated HSCs [37]. CK1δ/ε inhibition reduced TGF-β-induced HSC activation markers in both cellular and organoid systems in those in vitro studies. Fibrosis reciprocally suppresses *BMAL1* expression in activated HSCs in the same experimental models. These findings collectively suggest a cycle in which clock disruption and profibrotic signaling mutually reinforce one another.

REV-ERBα has emerged as the clock protein with the most directly demonstrable pharmacological relevance to hepatic fibrosis in preclinical models. It is important to note at the outset that SR9009, one of the most widely used synthetic REV-ERB ligands, has documented REV-ERB-independent effects on cell viability and gene expression. Thomes and colleagues demonstrated that SR9009 suppresses HSC proliferation and fibrogenic marker expression through blockade of P70S6K phosphorylation in cell-based studies [96]. Wang and colleagues showed that the REV-ERBα agonist GSK4112 attenuates CCl4-induced fibrosis by suppressing NLRP3 inflammasome activation in animal models [97]. Griffett and colleagues confirmed anti-fibrotic effects of SR9009 in a diet-induced MASH animal model [98]. No human clinical trial has evaluated REV-ERBα agonism in liver fibrosis.

The disruption of circadian clock gene expression directly remodels extracellular matrix composition at the tissue level in animal models. CLOCK∆19 mutant mice exhibit significantly elevated baseline collagen deposition following CCl4 challenge [99]. Transcriptomic data integrated with ATAC-seq from quiescent HSCs identified a potential CLOCK regulome associated with HSC quiescence maintenance in that study. *Per2* deletion elevates *Col1a1* and *Timp1* expression in cholestatic mouse liver. Evidence from non-hepatic models further suggests clock involvement in matrix remodeling. CLOCK directly drives *Timp3* rhythmic expression in human keratinocytes, and *Bmal1* deletion abolishes *Mmp2* and *Mmp9* activity rhythms in vascular tissue [100,101]. Those findings derive from skin and vascular systems rather than liver. Their applicability to the fibrotic hepatic environment therefore represents an inferential extrapolation rather than direct evidence.

The Kupffer cell circadian program contributes independently to fibrotic microenvironment remodeling. The relevant experimental evidence was established primarily in peritoneal macrophages and bone marrow-derived macrophages. Direct evidence in Kupffer cells specifically remains limited, and that constraint applies to all findings described in this paragraph. Pourcet and colleagues demonstrated that REV-ERBα controls the diurnal oscillation of NLRP3 inflammasome expression in hepatic macrophages, constraining the temporal pattern of IL-1β and IL-18 secretion [102]. Liu and colleagues established that myeloid BMAL1 drives the time-of-day dependence of hepatic inflammatory injury through direct regulation of Junb transcription in animal models [103]. Li and colleagues reported that circadian disruption in HFD-fed mice activates the RIPK1-RIPK3-MLKL necroptosis pathway in Kupffer cells with progressive collagen deposition [104]. That finding represents a single study and independent replication has not yet been published. It should therefore be regarded as a hypothesis-generating observation rather than an established mechanistic feature of fibrosis-associated clock disruption.

#### 3.3.2. Ethanol-Induced Disruption of Hepatic Clock Output and Metabolic Rhythms

Chronic ethanol exposure directly suppresses the oscillatory amplitude of core hepatic clock genes in animal models, while leaving the suprachiasmatic nucleus substantially intact. Alcohol feeding dampens diurnal oscillations of *Bmal1*, *Clock*, *Cry1*, *Cry2*, *Per1*, and *Per2* in mouse liver. Downstream clock-controlled output genes including *Dbp*, *Hlf*, and *Nr1d1* are equally attenuated in those animal studies. This liver-specific suppression is also detectable in human peripheral blood mononuclear cells. In alcohol-dependent patients, baseline expression of all six core clock genes in PBMCs was markedly lower than in controls. Only limited restoration was observed after one week of detoxification. Those PBMC findings provide directional human-level evidence for ethanol-induced clock suppression. They do not directly reflect hepatic clock gene status. Tissue-specific differences in circadian gene regulation mean that PBMC measurements should be understood as an accessible surrogate rather than a substitute for liver tissue data.

The mechanisms underlying hepatic clock suppression converge on two parallel axes established in animal and cell-based studies. The first axis concerns ethanol-induced disruption of the NAD^+^/NADH oscillatory cycle. This impairs SIRT1-mediated deacetylation of BMAL1 and PER2 in experimental systems. Acute ethanol exposure decreases hepatic SIRT1 levels and AMPKα phosphorylation. It simultaneously elevates BMAL1 and PER2 protein abundance in a pattern consistent with impaired deacetylation-dependent protein turnover [105]. The second axis involves CYP2E1-derived reactive oxygen species suppressing CLOCK:BMAL1 transcriptional activity at E-box regulatory elements in cell-based models. These two axes act in concert as parallel contributors to clock suppression. They should not be understood as sequential steps in a single upstream cascade.

BMAL1 has emerged as the central node through which ethanol-induced clock disruption translates into hepatic metabolic injury. In animal models, ethanol exposure impairs BMAL1-dependent temporal coordination between de novo lipogenesis and fatty acid oxidation, resulting in pathological lipid accumulation. Liver-specific *Bmal1* knockout mice fed ethanol developed more severe steatosis than wild-type controls in those animal experiments, confirming that intact BMAL1 function is hepatoprotective in the context of alcohol exposure [41]. Valcin and colleagues showed in ethanol-fed mice that alcohol feeding induced arrhythmicity in *Cry1*, *Cry2*, and *Nfil3*, dampened *Bmal1* and *Dbp* rhythms, and phase-advanced *Nr1d1* expression [40]. The resulting triglyceride lipidome remodeling involved altered fatty acid chain length and saturation profiles detectable only through lipidomic analysis. Two pharmacological studies confirm that BMAL1 is a viable therapeutic target in preclinical ALD models. Nobiletin failed to protect *Bmal1*-deficient mice from ethanol-induced liver injury while protecting wild-type animals [106]. Physcion directly occupied the BMAL1 active pocket, reversed clock gene suppression, and lost its hepatoprotective effect upon *Bmal1* siRNA knockdown in animal experiments [107]. Whether these pharmacological findings translate to human ALD has not been tested in clinical studies.

Ethanol also uncouples gut microbiota circadian oscillations from hepatic clock output through intestinal epithelial clock disruption. CYP2E1-derived oxidative stress in the intestinal epithelium drives PER2 induction through a PKA-CREB signaling cascade [108]. Among the limited human evidence available in this field, Swanson and colleagues showed that moderate alcohol consumption produced intestinal hyperpermeability and elevated plasma LPS in night workers. This effect was not observed in day workers with intact circadian alignment [109]. That human study provides directional evidence that circadian misalignment amplifies ethanol-induced intestinal barrier dysfunction. Its cross-sectional design does not permit causal inference, and tissue-level clock-microbiota coupling cannot be assessed from plasma measurements alone.

Loss of rhythmic microbial metabolite delivery further compounds the direct hepatic clock suppression caused by ethanol. In alcohol-dependent patients, only 0.66% of gut microbial operational taxonomic units oscillated diurnally, compared with 1.68% in healthy controls [110]. That clinical finding provides direct human evidence for the near-complete abolition of microbiota compositional rhythms in ALD. This reduction in oscillatory microbiota likely diminishes the circadian supply of SCFAs and secondary bile acids that normally entrain hepatic clock output [111]. Zhang and colleagues demonstrated in animal models that ellagic acid restored hepatic NPAS2 expression through microbiota reshaping, and that germ-free and antibiotic-treated mice lost this rescue effect entirely [112]. The sole genome-wide time-resolved multi-omics study in ethanol-treated liver was conducted by Gaucher and colleagues, who revealed distinct metabolic reprogramming between acute and chronic ethanol exposure [113]. That study identified rewired acetyl-CoA metabolism and altered BMAL1 acetylation patterns detectable only through combined proteomic and acetylome profiling. Beyond that single study, no time-resolved phosphoproteomics and no metabolomics with diurnal resolution have been performed in any ALD model.

#### 3.3.3. Mechanistic Inference and Evidence Gaps in Cholestatic Liver Disease

The relationship between the circadian clock and bile acid homeostasis operates in both directions. Clock gene loss produces elevated serum bile acids and spontaneous hepatic cholestasis through arrhythmic *Cyp7a1* and *Slc10a1* expression in animal models. *Per2* deletion further sensitizes the liver to bile duct ligation-induced fibrotic injury in those experimental systems. The reverse relationship is supported by two convergent but mechanistically independent lines of evidence. Powell and colleagues demonstrated that lithocholic acid stabilizes CRY2 by modulating CK1δ and CK1ε phosphatase feedback in human intestinal epithelial cells [68]. That mechanism was established specifically in intestinal rather than hepatic or biliary tissue. Whether equivalent bile acid-driven clock modulation operates in cholangiocytes or hepatocytes remains to be directly tested. Cholestasis-associated inflammatory cytokines impose a parallel and independent suppressive route on clock gene expression. These two pathways should be understood as distinct contributors to circadian disruption in cholestatic disease rather than sequential steps in a single cascade. Parenteral nutrition-associated cholestatic injury attenuates REV-ERBα, BMAL1, and PER2 protein levels and produces measurable hepatic-ileal circadian discordance in mouse models [114].

The cholangiocyte is the cellular target most directly relevant to PBC and PSC pathology. Its intrinsic clock program has received no time-resolved investigation. The available evidence derives almost entirely from melatonin receptor-mediated pharmacological interventions in rodent bile duct ligation models rather than from direct time-resolved profiling of cholangiocyte circadian oscillations. Sustained melatonin treatment in the Mdr2-knockout PSC model reduced biliary proliferation, senescence, and fibrosis over three months [115]. Pinealectomy or continuous light exposure exacerbated bile duct ligation-induced biliary injury through clock gene dysregulation and miR-200b induction in animal experiments [116]. These studies confirm that cholangiocytes possess a functional clock gene apparatus that is modulated during cholestatic injury. The intrinsic oscillatory dynamics of the biliary epithelial clock therefore remain uncharacterized at the mechanistic level. In PBC patient tissue, *AANAT* is substantially suppressed in cholangiocytes, suggesting impaired local circadian signaling capacity in the diseased biliary epithelium [117].

Clinical observations confirm that circadian disruption is a measurable phenotype in human cholestatic disease, though the available studies are exploratory in nature. A pilot study of morning bright light therapy in PBC demonstrated partial restoration of sleep timing and a trend toward improved circadian melatonin rhythmicity in a small sample [118]. A comparative study of non-cirrhotic PBC and PSC patients identified differences in fatigue character and chronotype between the two conditions [119]. These clinical observations describe phenotypic patterns rather than molecular clock mechanisms.

Three recently published datasets offer a near-term opportunity to begin addressing this gap through computational reanalysis of existing resources. Andrews and colleagues generated a combined single-cell and spatial transcriptomic atlas of PSC liver encompassing more than 47,000 cells profiled by Visium and GeoMx platforms [120]. Jin and colleagues profiled PBC patient liver at single-cell resolution across hepatocyte, cholangiocyte, and immune cell populations [121]. Li and colleagues combined single-cell sequencing, proteomics, and spatial transcriptomics to characterize cholangiocyte subpopulations in PBC in detail [122]. None of these datasets was designed with circadian sampling in mind. Reanalysis would therefore be limited to cross-sectional clock gene expression patterns rather than rhythmicity assessment. The distribution of available omics evidence, evidence depth, and principal research gaps across all disease categories are summarized in Table 3.

## 4. Translational Perspectives on Circadian Medicine in Liver Disease

### 4.1. Time-Resolved Biomarker Development and Sampling Standardization

Multiple candidate circadian biomarkers have emerged across the liver disease spectrum, including rhythmic plasma bile acids, branched-chain amino acid oscillations, and PBMC clock gene expression profiles. Their clinical utility remains unrealized because almost no published biomarker study has standardized sample collection time. Without temporal standardization, phase-shifted signals across individuals cancel during group-level analysis, producing artifactual arrhythmicity that obscures genuine phase displacement. Transcriptomic phase inference from peripheral blood offers a practical route toward time-resolved biomarker assessment. TimeSignature infers circadian time from two whole-blood transcriptomic draws separated by at least twelve hours [123,124]. TimeMachine reduces this to a single PBMC draw using a 135-gene phase indicator set [125]. Standardizing collection relative to habitual wake time rather than absolute clock time is more robust across individuals with different chronotypes. Applying these tools to existing MAFLD, viral hepatitis, and fibrosis biobanks requires no new tissue collection. Whether phase-aware reanalysis can recover meaningful rhythmic signals from existing datasets remains to be demonstrated.

Translating these methodological advances into clinically actionable biomarker studies will require attention to several practical requirements that current research designs have not addressed. First, sampling time should be standardized relative to each participant’s habitual wake time rather than absolute clock time. Internal circadian phase varies substantially across individuals with different chronotypes and sleep schedules. Second, PBMC-derived phase estimates should be interpreted as surrogate indicators of systemic circadian phase rather than direct measures of hepatic clock status. Tissue-specific differences in circadian gene regulation mean that hepatic and peripheral clock states can diverge substantially, particularly in the context of liver disease. Third, future biomarker studies should stratify participants by disease stage and etiology. The evidence reviewed here indicates that clock dysregulation patterns differ between MAFLD, viral hepatitis, fibrosis, and cholestatic disease, and pooling across these categories without stratification will obscure disease-specific rhythmic signatures. Fourth, intervention trials targeting circadian pathways should pre-specify circadian rhythm endpoints rather than treating them as secondary or exploratory outcomes. Without pre-specified temporal endpoints, the circadian efficacy of clock-directed interventions cannot be formally evaluated regardless of the mechanistic rationale underlying the treatment.

### 4.2. Circadian Targets for Therapeutic Intervention Across the Disease Spectrum

The mechanistic evidence reviewed here converges on three therapeutic axes addressing circadian dysfunction at distinct molecular levels. REV-ERBα agonism is the most extensively supported clock-directed strategy across multiple disease contexts. In hepatic steatosis, REV-ERBα governs rhythmic suppression of CYP7A1 and temporal organization of cholesterol-to-bile acid conversion. In liver fibrosis, its pharmacological activation attenuates NLRP3 inflammasome gating in hepatic macrophages and reduces collagen deposition in preclinical models. Synthetic ligands SR9009 and SR9011 have demonstrated efficacy across steatohepatitis, fibrosis, and metabolic dysregulation models. SR9009 carries documented REV-ERB-independent effects on cell viability and gene expression, as demonstrated in REV-ERBα/β double-knockout cells [98]. Mechanistic conclusions from SR9009 studies should therefore not be attributed solely to REV-ERBα activation. No human clinical trial has yet evaluated REV-ERBα agonism in any liver disease indication.

A second axis concerns chronopharmacological optimization of existing liver-directed drugs. FXR and its downstream mediator SHP exhibit diurnal expression rhythms gated through interactions with REV-ERBα and related nuclear receptor networks. This proposition should be understood as a hypothesis-driven research direction rather than an established pharmacological principle. The efficacy of FXR agonists such as obeticholic acid may depend in part on the circadian phase of administration. That dependency has not been tested in any time-resolved study. Incorporating dosing time as a covariate in future FXR agonist trials requires no additional pharmacological development and represents a low-cost design modification worth prospective evaluation.

The third axis involves restoration of the NAD^+^ oscillatory system as an upstream driver of clock protein stability. NAD^+^ levels decline in MAFLD and MASH, impairing SIRT1-mediated deacetylation of BMAL1 and PER2 and destabilizing the primary oscillatory loop. Levine and colleagues demonstrated in aged mice that oral nicotinamide riboside supplementation restored BMAL1 chromatin binding genome-wide through SIRT1-dependent PER2 deacetylation [24]. In healthy middle-aged and older adults, nicotinamide riboside at 500 mg daily for six weeks is well tolerated and produces meaningful elevation of whole-blood NAD^+^ concentrations [126]. A double-blind placebo-controlled trial of combined nicotinamide riboside and pterostilbene in biopsy-proven MAFLD patients demonstrated reductions in hepatic inflammation markers [127]. That trial was not designed to assess circadian rhythm endpoints. Whether NAD^+^ repletion can restore oscillatory amplitude in human liver disease tissue therefore remains untested.

Several limitations constrain the translational reach of all three axes. Human time-resolved liver tissue data are nearly absent across all disease categories reviewed here. Ethical constraints on serial liver biopsies, the logistical demands of nocturnal sampling, and the absence of circadian endpoints in existing trial designs collectively account for this gap. REV-ERBα agonism has not advanced beyond preclinical models in any liver disease indication. NAD^+^ intervention studies have not been designed around circadian endpoints, meaning their circadian efficacy cannot be inferred from existing trial results. Addressing these gaps requires prospective studies with temporally designed sampling, etiology-stratified cohorts, and pre-specified circadian endpoints.

## 5. Conclusions

The evidence reviewed here supports the proposition that circadian clock dysregulation constitutes a shared pathological axis across the chronic liver disease spectrum. Four molecular features recur across disease contexts. BMAL1 functional downregulation, REV-ERBα output attenuation, NAD^+^ amplitude reduction, and gut–liver axis desynchronization represent partially overlapping recurring motifs rather than a uniform mechanistic signature. Together, they form a proposed inferential framework for hepatic circadian failure. This framework is not an established pan-disease mechanism. The upstream drivers and the strength of supporting evidence vary considerably across disease categories and omics layers. The multi-omics evidence base is distributed unevenly. Transcriptomic and epigenomic data are comparatively mature for MAFLD and HCV hepatitis. In HCC, evidence spanning transcriptional dysregulation, post-translational clock protein remodeling, and metabolic reprogramming provides the most multi-layered picture currently available. ALD has a single genome-wide time-resolved study. That study requires independent replication before its findings can be generalized. HBV-related disease and cholestatic liver disease lack multi-omics data entirely. Human PBC and PSC liver tissue remains unmeasured at the molecular clock level. The near-complete absence of temporally resolved human tissue data remains the principal barrier across all disease categories. Apparent discrepancies between animal and human findings, most evident in the BMAL1-fibrosis relationship, most plausibly reflect model design differences rather than fundamental interspecies divergence. Circadian phase inference algorithms provide a methodological foundation for addressing this gap. Prospective studies with temporally designed sampling, etiology-stratified cohorts, and pre-specified circadian endpoints represent the most direct path toward clinically actionable circadian hepatology.

## Figures and Tables

**Table 1:** Clock gene expression alterations across the chronic liver disease spectrum.

Disease Context	Downregulated Clock Components	Upregulated Clock Components	References
MAFLD	*BMAL1*, *PER2*, *PER3*, *CRY2*, *NR1D2* (REV-ERBβ), *RORA*	—	[29,30]
HCV Hepatitis	*PER2*, *CRY2*, *RORA*, *BMAL1*	—	[31]
HBV Hepatitis	*PER1*, *PER2*, *PER3*, *CRY2*	*CLOCK*, *BMAL1*, *CRY1*	[32]
HCC	*BMAL1*, *PER1*, *PER2*, *CRY2*, *RORA*	*TIMELESS*, *NPAS2*	[33,34]
ALD	*BMAL1*, *CLOCK*, *PER1*, *PER2*, *CRY1*, *CRY2*, *DBP*, *NR1D1* (phase-advanced)	—	[35]
Liver Fibrosis	*BMAL1*, *NR1D1*	—	[36,37]
Cholestatic Disease (PBC and PSC)	*NR1D1*, *BMAL1*, *PER2*	—	[38,39]

**Table 2:** Gut microbiota-derived rhythmic signals and their hepatic clock targets.

Signal Class	Representative Molecules	Hepatic Molecular Target	Mechanistic Basis	Evidence Level	References
Short-Chain Fatty Acids	Butyrate, propionate	BMAL1 chromatin accessibility	Cyclic HDAC inhibition via rhythmic SCFA production	Limited (human peripheral blood RCT only; no hepatic tissue data)	[67]
Secondary Bile Acids	Lithocholic acid, deoxycholic acid, BSH-modified bile acid pool	DBP, *PER1*, *PER2* transcription; CRY2 stability via CK1δ/ε feedback	BSH-dependent bile acid pool modification and CRY2 kinase feedback	Limited (human colonic cell lines only; no hepatic tissue data)	[68]
Tryptophan-Derived Metabolites	Indole derivatives	CLOCK:BMAL1 E-box activity via AhR–ARNT competition	AhR–ARNT structural competition at E-box elements	Preclinical only (mouse models and cell-based systems)	[65,66]

**Table 3:** Multi-omics evidence coverage, depth, and principal research gaps by disease category.

Disease Category	Omics Evidence and Coverage	Principal Evidence Gaps
MAFLD	Transcriptomics, epigenomics, targeted phosphoproteomics, lipidomics, and bile acid metabolomics	No time-resolved phosphoproteomic study in any MAFLD model or human cohort
HCV Hepatitis	Time-resolved transcriptomics and epigenomics in human liver chimeric model; human biopsy cohort validation	No proteomic or metabolomic data at any disease stage
HBV Hepatitis	Targeted transcript-level gene expression studies in small cohorts	All findings at transcript level; no time-resolved or multi-omics data at any disease stage
HCC	Transcriptomics, epigenomics, targeted PTM, single-cell RNA-seq, and spatial transcriptomics	No global phosphoproteomic, acetylome, or ubiquitome profiling in HCC tissue
ALD	Targeted transcriptomics in human PBMC and mouse liver; one genome-wide time-resolved multi-omics study	No time-resolved human liver data; single multi-omics study requires replication
Liver Fibrosis	Transcriptomics, targeted proteomics of HSC markers, and ATAC-seq from quiescent HSCs	No time-resolved MMP or TIMP profiling in fibrotic liver
Cholestatic Disease (PBC and PSC)	Rodent pharmacological studies; clinical phenotyping; existing scRNA-seq and spatial datasets available for reanalysis	Clock gene expression unmeasured in human PBC or PSC liver tissue

## Data Availability

No new data were created or analyzed in this study. Data sharing is not applicable to this article.

## Abbreviations

The following abbreviations are used in this manuscript:

AhR	Aryl hydrocarbon receptor
ALD	Alcoholic liver disease
AMPK	AMP-activated protein kinase
ARNT	Aryl hydrocarbon receptor nuclear translocator
ATM	Ataxia telangiectasia mutated kinase
BHB	Beta-hydroxybutyrate
BMAL1	Brain and muscle ARNT-like protein 1
BSH	Bile salt hydrolase
CK1	Casein kinase 1
CK2	Casein kinase 2
CLOCK	Circadian locomotor output cycles kaput
CRY	Cryptochrome
CYP7A1	Cytochrome P450 family 7 subfamily A member 1
DBP	D-box binding PAR bZIP transcription factor
EZH2	Enhancer of zeste homolog 2
FBXL3	F-box and leucine-rich repeat protein 3
FXR	Farnesoid X receptor
HBV	Hepatitis B virus
HBx	Hepatitis B virus X protein
HCC	Hepatocellular carcinoma
HCV	Hepatitis C virus
HDAC	Histone deacetylase
HIF-1α	Hypoxia-inducible factor 1 alpha
HSC	Hepatic stellate cell
IGF1R	Insulin-like growth factor 1 receptor
MAFLD	Metabolic dysfunction-associated fatty liver disease
MMP	Matrix metalloproteinase
mTOR	Mechanistic target of rapamycin
NAD^+^	Nicotinamide adenine dinucleotide
NF-κB	Nuclear factor kappa-light-chain-enhancer of activated B cells
NLRP3	NLR family pyrin domain containing 3
NPAS2	Neuronal PAS domain protein 2
NR	Nicotinamide riboside
NR1D1	Nuclear receptor subfamily 1 group D member 1
NR1D2	Nuclear receptor subfamily 1 group D member 2
NTCP	Sodium taurocholate cotransporting polypeptide
PAS	Per-Arnt-Sim domain
PBC	Primary biliary cholangitis
PBMC	Peripheral blood mononuclear cell
PER	Period circadian regulator
PPARα	Peroxisome proliferator-activated receptor alpha
PRPS	Phosphoribosyl pyrophosphate synthetase
PSC	Primary sclerosing cholangitis
PTM	Post-translational modification
REV-ERBα	Nuclear receptor subfamily 1 group D member 1 (NR1D1)
RORα	RAR-related orphan receptor alpha
ROS	Reactive oxygen species
SCN	Suprachiasmatic nucleus
SCFA	Short-chain fatty acid
SIRT1	Sirtuin 1
SREBP1c	Sterol regulatory element-binding protein 1c
TGF-β	Transforming growth factor beta
TGR5	Takeda G protein-coupled receptor 5
TIMP	Tissue inhibitor of metalloproteinase
TNF-α	Tumor necrosis factor alpha
ZT	Zeitgeber time

## References

[1] Tahara Y., Shibata S. (2016). Circadian Rhythms of Liver Physiology and Disease: Experimental and Clinical Evidence. Nat. Rev. Gastroenterol. Hepatol..

[2] Takahashi J.S. (2017). Transcriptional Architecture of the Mammalian Circadian Clock. Nat. Rev. Genet..

[3] Panda S. (2016). Circadian Physiology of Metabolism. Science.

[4] Guan D., Xiong Y., Trinh T.M., Xiao Y., Hu W., Jiang C., Dierickx P., Jang C., Rabinowitz J.D., Lazar M.A. (2020). The Hepatocyte Clock and Feeding Control Chronophysiology of Multiple Liver Cell Types. Science.

[5] Greco C.M., Koronowski K.B., Smith J.G., Shi J., Kunderfranco P., Carriero R., Chen S., Samad M., Welz P.-S., Zinna V.M. (2021). Integration of Feeding Behavior by the Liver Circadian Clock Reveals Network Dependency of Metabolic Rhythms. Sci. Adv..

[6] Koronowski K.B., Kinouchi K., Welz P.-S., Smith J.G., Zinna V.M., Shi J., Samad M., Chen S., Magnan C.N., Kinchen J.M. (2019). Defining the Independence of the Liver Circadian Clock. Cell.

[7] Pickel L., Sung H.-K. (2020). Feeding Rhythms and the Circadian Regulation of Metabolism. Front. Nutr..

[8] Bolshette N., Ibrahim H., Reinke H., Asher G. (2023). Circadian Regulation of Liver Function: From Molecular Mechanisms to Disease Pathophysiology. Nat. Rev. Gastroenterol. Hepatol..

[9] Mukherji A., Bailey S.M., Staels B., Baumert T.F. (2019). The Circadian Clock and Liver Function in Health and Disease. J. Hepatol..

[10] Gu W., Li T., Huang Y., Wang R., Zhang L., Chen R., Li R., Liu C. (2024). Metabolic Profile and Lipid Metabolism Phenotype in Mice with Conditional Deletion of Hepatic BMAL1. Int. J. Mol. Sci..

[11] De Assis L.V.M., Demir M., Oster H. (2023). The Role of the Circadian Clock in the Development, Progression, and Treatment of Non-alcoholic Fatty Liver Disease. Acta Physiol..

[12] Bailey S.M. (2018). Emerging Role of Circadian Clock Disruption in Alcohol-Induced Liver Disease. Am. J. Physiol. Gastrointest. Liver Physiol..

[13] Chen M., Lin Y., Dang Y., Xiao Y., Zhang F., Sun G., Jiang X., Zhang L., Du J., Duan S. (2023). Reprogramming of Rhythmic Liver Metabolism by Intestinal Clock. J. Hepatol..

[14] Mukherji A., Jühling F., Simanjuntak Y., Crouchet E., Del Zompo F., Teraoka Y., Haller A., Baltzinger P., Paritala S., Rasha F. (2024). An Atlas of the Human Liver Diurnal Transcriptome and Its Perturbation by Hepatitis C Virus Infection. Nat. Commun..

[15] Zhuang X., Forde D., Tsukuda S., D’Arienzo V., Mailly L., Harris J.M., Wing P.A.C., Borrmann H., Schilling M., Magri A. (2021). Circadian Control of Hepatitis B Virus Replication. Nat. Commun..

[16] Qu M., Zhang G., Qu H., Vu A., Wu R., Tsukamoto H., Jia Z., Huang W., Lenz H.-J., Rich J.N. (2023). Circadian Regulator BMAL1::CLOCK Promotes Cell Proliferation in Hepatocellular Carcinoma by Controlling Apoptosis and Cell Cycle. Proc. Natl. Acad. Sci. USA.

[17] Liu T., Wang Z., Ye L., Duan Y., Jiang H., He H., Xiao L., Wu Q., Xia Y., Yang M. (2023). Nucleus-Exported CLOCK Acetylates PRPS to Promote de Novo Nucleotide Synthesis and Liver Tumour Growth. Nat. Cell Biol..

[18] Mauvoisin D., Wang J., Jouffe C., Martin E., Atger F., Waridel P., Quadroni M., Gachon F., Naef F. (2014). Circadian Clock-Dependent and -Independent Rhythmic Proteomes Implement Distinct Diurnal Functions in Mouse Liver. Proc. Natl. Acad. Sci. USA.

[19] Robles M.S., Humphrey S.J., Mann M. (2017). Phosphorylation Is a Central Mechanism for Circadian Control of Metabolism and Physiology. Cell Metab..

[20] Mauvoisin D., Atger F., Dayon L., Núñez Galindo A., Wang J., Martin E., Da Silva L., Montoliu I., Collino S., Martin F.-P. (2017). Circadian and Feeding Rhythms Orchestrate the Diurnal Liver Acetylome. Cell Rep..

[21] Ray S., Valekunja U.K., Stangherlin A., Howell S.A., Snijders A.P., Damodaran G., Reddy A.B. (2020). Circadian Rhythms in the Absence of the Clock Gene *Bmal1*. Science.

[22] Martini T., Naef F., Tchorz J.S. (2023). Spatiotemporal Metabolic Liver Zonation and Consequences on Pathophysiology. Annu. Rev. Pathol..

[23] Asher G., Zhu B. (2023). Beyond Circadian Rhythms: Emerging Roles of Ultradian Rhythms in Control of Liver Functions. Hepatology.

[24] Levine D.C., Hong H., Weidemann B.J., Ramsey K.M., Affinati A.H., Schmidt M.S., Cedernaes J., Omura C., Braun R., Lee C. (2020). NAD+ Controls Circadian Reprogramming through PER2 Nuclear Translocation to Counter Aging. Mol. Cell.

[25] Fu Z., Kim H., Morse P.T., Lu M.-J., Hüttemann M., Cambronne X.A., Zhang K., Zhang R. (2022). The Mitochondrial NAD+ Transporter SLC25A51 Is a Fasting-Induced Gene Affecting SIRT3 Functions. Metabolism.

[26] Ness-Cohn E., Allada R., Braun R. (2021). Comment on “Circadian Rhythms in the Absence of the Clock Gene *Bmal1*”. Science.

[27] Eckel-Mahan K.L., Patel V.R., Mohney R.P., Vignola K.S., Baldi P., Sassone-Corsi P. (2012). Coordination of the Transcriptome and Metabolome by the Circadian Clock. Proc. Natl. Acad. Sci. USA.

[28] Droin C., Kholtei J.E., Bahar Halpern K., Hurni C., Rozenberg M., Muvkadi S., Itzkovitz S., Naef F. (2021). Space-Time Logic of Liver Gene Expression at Sub-Lobular Scale. Nat. Metab..

[29] Xie M., Tang Q., Nie J., Zhang C., Zhou X., Yu S., Sun J., Cheng X., Dong N., Hu Y. (2020). BMAL1-Downregulation Aggravates *Porphyromonas Gingivalis*-Induced Atherosclerosis by Encouraging Oxidative Stress. Circ. Res..

[30] Jouffe C., Weger B.D., Martin E., Atger F., Weger M., Gobet C., Ramnath D., Charpagne A., Morin-Rivron D., Powell E.E. (2022). Disruption of the Circadian Clock Component BMAL1 Elicits an Endocrine Adaption Impacting on Insulin Sensitivity and Liver Disease. Proc. Natl. Acad. Sci. USA.

[31] Horii R., Honda M., Shirasaki T., Shimakami T., Shimizu R., Yamanaka S., Murai K., Kawaguchi K., Arai K., Yamashita T. (2019). MicroRNA-10a Impairs Liver Metabolism in Hepatitis C Virus-Related Cirrhosis Through Deregulation of the Circadian Clock Gene Brain and Muscle Aryl Hydrocarbon Receptor Nuclear Translocator-Like 1. Hepatol. Commun..

[32] Yang S.-L., Yu C., Jiang J.-X., Liu L.-P., Fang X., Wu C. (2014). Hepatitis B Virus X Protein Disrupts the Balance of the Expression of Circadian Rhythm Genes in Hepatocellular Carcinoma. Oncol. Lett..

[33] Li H., Lu Y.-F., Chen H., Liu J. (2017). Dysregulation of Metallothionein and Circadian Genes in Human Hepatocellular Carcinoma. Chronobiol. Int..

[34] Fekry B., Ribas-Latre A., Baumgartner C., Deans J.R., Kwok C., Patel P., Fu L., Berdeaux R., Sun K., Kolonin M.G. (2018). Incompatibility of the Circadian Protein BMAL1 and HNF4α in Hepatocellular Carcinoma. Nat. Commun..

[35] Udoh U., Valcin J., Gamble K., Bailey S. (2015). The Molecular Circadian Clock and Alcohol-Induced Liver Injury. Biomolecules.

[36] Crouchet E., Dachraoui M., Jühling F., Roehlen N., Oudot M.A., Durand S.C., Ponsolles C., Gadenne C., Meiss-Heydmann L., Moehlin J. (2025). Targeting the Liver Clock Improves Fibrosis by Restoring TGF-β Signaling. J. Hepatol..

[37] Johanns M., Berthier A., Vandel J., Courquet S., Dubois-Chevalier J., Vinod M., Very N., Zummo F.P., Bobowski-Gérard M., Toma G. (2025). The Circadian Clock Controls Hepatic Stellate Cell Activation in Liver Fibrosis via a BMAL1/CK1ε/REV-ERBα/Transgelin Signaling Pathway. bioRxiv.

[38] Shearn C.T., Anderson A.L., Devereaux M.W., El Kasmi K.C., Orlicky D.J., Sokol R.J. (2023). Expression of Circadian Regulatory Genes Is Dysregulated by Increased Cytokine Production in Mice Subjected to Concomitant Intestinal Injury and Parenteral Nutrition. PLoS ONE.

[39] Zhou L., Yan M., Luo Q., Qiu W., Guo Y.-R., Guo X.-Q., Yu H.-B., Huo J.-R., Feng Y.-L., Wang D.-P. (2025). Elevated Bile Acids Induce Circadian Rhythm Sleep Disorders in Chronic Liver Diseases. Cell. Mol. Gastroenterol. Hepatol..

[40] Valcin J.A., Udoh U.S., Swain T.M., Andringa K.K., Patel C.R., Al Diffalha S., Baker P.R.S., Gamble K.L., Bailey S.M. (2020). Alcohol and Liver Clock Disruption Increase Small Droplet Macrosteatosis, Alter Lipid Metabolism and Clock Gene mRNA Rhythms, and Remodel the Triglyceride Lipidome in Mouse Liver. Front. Physiol..

[41] Zhang D., Tong X., Nelson B.B., Jin E., Sit J., Charney N., Yang M., Omary M.B., Yin L. (2018). The Hepatic BMAL1/AKT/Lipogenesis Axis Protects against Alcoholic Liver Disease in Mice via Promoting PPARα Pathway. Hepatology.

[42] Larion S., Padgett C.A., Butcher J.T., Mintz J.D., Fulton D.J., Stepp D.W. (2022). The Biological Clock Enhancer Nobiletin Ameliorates Steatosis in Genetically Obese Mice by Restoring Aberrant Hepatic Circadian Rhythm. Am. J. Physiol. Gastrointest. Liver Physiol..

[43] Hong H.-K., Maury E., Ramsey K.M., Perelis M., Marcheva B., Omura C., Kobayashi Y., Guttridge D.C., Barish G.D., Bass J. (2018). Requirement for NF-κB in Maintenance of Molecular and Behavioral Circadian Rhythms in Mice. Genes. Dev..

[44] Shen Y., Endale M., Wang W., Morris A.R., Francey L.J., Harold R.L., Hammers D.W., Huo Z., Partch C.L., Hogenesch J.B. (2021). NF-κB Modifies the Mammalian Circadian Clock through Interaction with the Core Clock Protein BMAL1. PLoS Genet..

[45] Li F., Lin L., He Y., Sun G., Dong D., Wu B. (2022). BMAL1 Regulates *Propionibacterium Acnes*-Induced Skin Inflammation via REV-ERBα in Mice. Int. J. Biol. Sci..

[46] Duong H.A., Baba K., DeBruyne J.P., Davidson A.J., Ehlen C., Powell M., Tosini G. (2024). Environmental Circadian Disruption Re-Writes Liver Circadian Proteomes. Nat. Commun..

[47] Dadon-Freiberg M., Chapnik N., Froy O. (2021). REV-ERBα Alters Circadian Rhythms by Modulating mTOR Signaling. Mol. Cell. Endocrinol..

[48] Wattacheril J., Rose K.L., Hill S., Lanciault C., Murray C.R., Washington K., Williams B., English W., Spann M., Clements R. (2017). Non-alcoholic Fatty Liver Disease Phosphoproteomics: A Functional Piece of the Precision Puzzle. Hepatol. Res..

[49] Adamovich Y., Rousso-Noori L., Zwighaft Z., Neufeld-Cohen A., Golik M., Kraut-Cohen J., Wang M., Han X., Asher G. (2014). Circadian Clocks and Feeding Time Regulate the Oscillations and Levels of Hepatic Triglycerides. Cell Metab..

[50] Chaix A., Lin T., Le H.D., Chang M.W., Panda S. (2019). Time-Restricted Feeding Prevents Obesity and Metabolic Syndrome in Mice Lacking a Circadian Clock. Cell Metab..

[51] Mehus A.A., Rust B., Idso J.P., Hanson B., Zeng H., Yan L., Bukowski M.R., Picklo M.J. (2021). Time-Restricted Feeding Mice a High-Fat Diet Induces a Unique Lipidomic Profile. J. Nutr. Biochem..

[52] Huang R., Chen J., Zhou M., Xin H., Lam S.M., Jiang X., Li J., Deng F., Shui G., Zhang Z. (2023). Multi-Omics Profiling Reveals Rhythmic Liver Function Shaped by Meal Timing. Nat. Commun..

[53] Ferrell J.M., Chiang J.Y.L. (2015). Short-Term Circadian Disruption Impairs Bile Acid and Lipid Homeostasis in Mice. Cell Mol. Gastroenterol. Hepatol..

[54] Eggink H.M., Oosterman J.E., de Goede P., de Vries E.M., Foppen E., Koehorst M., Groen A.K., Boelen A., Romijn J.A., la Fleur S.E. (2017). Complex Interaction between Circadian Rhythm and Diet on Bile Acid Homeostasis in Male Rats. Chronobiol. Int..

[55] Kettner N.M., Voicu H., Finegold M.J., Coarfa C., Sreekumar A., Putluri N., Katchy C.A., Lee C., Moore D.D., Fu L. (2016). Circadian Homeostasis of Liver Metabolism Suppresses Hepatocarcinogenesis. Cancer Cell.

[56] Cal-Kayitmazbatir S., Kulkoyluoglu-Cotul E., Growe J., Selby C.P., Rhoades S.D., Malik D., Oner H., Asimgil H., Francey L.J., Sancar A. (2021). CRY1-CBS Binding Regulates Circadian Clock Function and Metabolism. FEBS J..

[57] Thaiss C.A., Zeevi D., Levy M., Zilberman-Schapira G., Suez J., Tengeler A.C., Abramson L., Katz M.N., Korem T., Zmora N. (2014). Transkingdom Control of Microbiota Diurnal Oscillations Promotes Metabolic Homeostasis. Cell.

[58] Thaiss C.A., Levy M., Korem T., Dohnalová L., Shapiro H., Jaitin D.A., David E., Winter D.R., Gury-BenAri M., Tatirovsky E. (2016). Microbiota Diurnal Rhythmicity Programs Host Transcriptome Oscillations. Cell.

[59] Heddes M., Altaha B., Niu Y., Reitmeier S., Kleigrewe K., Haller D., Kiessling S. (2022). The Intestinal Clock Drives the Microbiome to Maintain Gastrointestinal Homeostasis. Nat. Commun..

[60] Segers A., Desmet L., Thijs T., Verbeke K., Tack J., Depoortere I. (2019). The Circadian Clock Regulates the Diurnal Levels of Microbial Short-chain Fatty Acids and Their Rhythmic Effects on Colon Contractility in Mice. Acta Physiol..

[61] Tognini P., Murakami M., Liu Y., Eckel-Mahan K.L., Newman J.C., Verdin E., Baldi P., Sassone-Corsi P. (2017). Distinct Circadian Signatures in Liver and Gut Clocks Revealed by Ketogenic Diet. Cell Metab..

[62] Tahara Y., Yamazaki M., Sukigara H., Motohashi H., Sasaki H., Miyakawa H., Haraguchi A., Ikeda Y., Fukuda S., Shibata S. (2018). Gut Microbiota-Derived Short Chain Fatty Acids Induce Circadian Clock Entrainment in Mouse Peripheral Tissue. Sci. Rep..

[63] Yao L., Seaton S.C., Ndousse-Fetter S., Adhikari A.A., DiBenedetto N., Mina A.I., Banks A.S., Bry L., Devlin A.S. (2018). A Selective Gut Bacterial Bile Salt Hydrolase Alters Host Metabolism. eLife.

[64] Han L., Xu R., Conwell A.N., Takahashi S., Parasar B., Chang P.V. (2024). Bile Salt Hydrolase Activity-Based Probes for Monitoring Gut Microbial Bile Acid Metabolism. ChemBioChem.

[65] Corrada D., Soshilov A.A., Denison M.S., Bonati L. (2016). Deciphering Dimerization Modes of PAS Domains: Computational and Experimental Analyses of the AhR:ARNT Complex Reveal New Insights Into the Mechanisms of AhR Transformation. PLoS Comput. Biol..

[66] Gheorghe C.E., Ritz N.L., Martin J.A., Wardill H.R., Cryan J.F., Clarke G. (2021). Investigating Causality with Fecal Microbiota Transplantation in Rodents: Applications, Recommendations and Pitfalls. Gut Microbes.

[67] Firoozi D., Masoumi S.J., Mohammad-Kazem Hosseini Asl S., Labbe A., Razeghian-Jahromi I., Fararouei M., Lankarani K.B., Dara M. (2024). Effects of Short-Chain Fatty Acid-Butyrate Supplementation on Expression of Circadian-Clock Genes, Sleep Quality, and Inflammation in Patients with Active Ulcerative Colitis: A Double-Blind Randomized Controlled Trial. Lipids Health Dis..

[68] Powell C.E., McSween A.M., Dohnalová L., Kim C.H., Eisert R.J., Sun Z.-Y.J., Seo H.-S., Marquardt V., Dhe-Paganon S., Thaiss C.A. (2026). Gut Microbiome-Produced Bile Acid Metabolite Lengthens the Circadian Period in Host Intestinal Cells. Proc. Natl. Acad. Sci. USA.

[69] Frazier K., Kambal A., Zale E.A., Pierre J.F., Hubert N., Miyoshi S., Miyoshi J., Ringus D.L., Harris D., Yang K. (2022). High-Fat Diet Disrupts REG3γ and Gut Microbial Rhythms Promoting Metabolic Dysfunction. Cell Host Microbe.

[70] Zhao D., Wang X., Liu H., Su M., Sun M., Zhang L., Ye H. (2024). Effect of Circadian Rhythm Change on Gut Microbiota and the Development of Nonalcoholic Fatty Liver Disease in Mice. Sleep. Med..

[71] Yang Z., Zarbl H., Kong B., Taylor R., Black K., Kipen H., Basaly V., Fang M., Guo G.L. (2024). Liver–Gut Axis Signaling Regulates Circadian Energy Metabolism in Shift Workers. FASEB J..

[72] Zhuang X., Magri A., Hill M., Lai A.G., Kumar A., Rambhatla S.B., Donald C.L., Lopez-Clavijo A.F., Rudge S., Pinnick K. (2019). The Circadian Clock Components BMAL1 and REV-ERBα Regulate Flavivirus Replication. Nat. Commun..

[73] Hamdane N., Jühling F., Crouchet E., El Saghire H., Thumann C., Oudot M.A., Bandiera S., Saviano A., Ponsolles C., Roca Suarez A.A. (2019). HCV-Induced Epigenetic Changes Associated with Liver Cancer Risk Persist After Sustained Virologic Response. Gastroenterology.

[74] Hlady R.A., Zhao X., El Khoury L.Y., Luna A., Pham K., Wu Q., Lee J., Pyrsopoulos N.T., Liu C., Robertson K.D. (2022). Interferon Drives HCV Scarring of the Epigenome and Creates Targetable Vulnerabilities Following Viral Clearance. Hepatology.

[75] Park J., Jang K.L. (2014). Hepatitis C Virus Represses E-Cadherin Expression via DNA Methylation to Induce Epithelial to Mesenchymal Transition in Human Hepatocytes. Biochem. Biophys. Res. Commun..

[76] Yuan P., Yang T., Mu J., Zhao J., Yang Y., Yan Z., Hou Y., Chen C., Xing J., Zhang H. (2020). Circadian Clock Gene NPAS2 Promotes Reprogramming of Glucose Metabolism in Hepatocellular Carcinoma Cells. Cancer Lett..

[77] Wang X., He M., Zhou L., Chen W. (2025). EZH2 Expression in Hepatocellular Carcinoma and Its Relationship with Circadian Rhythm-Related Genes. Sci. Rep..

[78] Yang Y., Yang T., Zhao Z., Zhang H., Yuan P., Wang G., Zhao Z., An J., Lyu Z., Xing J. (2022). Down-Regulation of BMAL1 by MiR-494-3p Promotes Hepatocellular Carcinoma Growth and Metastasis by Increasing GPAM-Mediated Lipid Biosynthesis. Int. J. Biol. Sci..

[79] Yang Y., Abdo A.N., Kawara H., Selby C.P., Sancar A. (2023). Preservation of Circadian Rhythm in Hepatocellular Cancer. J. Biol. Chem..

[80] Liang Y., Wang S., Huang X., Chai R., Tang Q., Yang R., Huang X., Wang X., Zheng K. (2021). Dysregulation of Circadian Clock Genes as Significant Clinic Factor in the Tumorigenesis of Hepatocellular Carcinoma. Comput. Math. Methods Med..

[81] Chan A.B., Parico G.C.G., Fribourgh J.L., Ibrahim L.H., Bollong M.J., Partch C.L., Lamia K.A. (2021). *CRY2* Missense Mutations Suppress P53 and Enhance Cell Growth. Proc. Natl. Acad. Sci. USA.

[82] Huyen V.T., Echizen K., Yamagishi R., Kumagai M., Nonaka Y., Kodama T., Ando T., Yano M., Takada N., Takasugi M. (2024). Regular Exercise Suppresses Steatosis-associated Liver Cancer Development by Degrading E2F1 and c-Myc via Circadian Gene Upregulation. Genes. Cells.

[83] Mteyrek A., Filipski E., Guettier C., Okyar A., Lévi F. (2016). Clock Gene *Per2* as a Controller of Liver Carcinogenesis. Oncotarget.

[84] Yuan P., Li J., Zhou F., Huang Q., Zhang J., Guo X., Lyu Z., Zhang H., Xing J. (2017). NPAS2 Promotes Cell Survival of Hepatocellular Carcinoma by Transactivating CDC25A. Cell Death Dis..

[85] Zhao X., Hirota T., Han X., Cho H., Chong L.-W., Lamia K., Liu S., Atkins A.R., Banayo E., Liddle C. (2016). Circadian Amplitude Regulation via FBXW7-Targeted REV-ERBα Degradation. Cell.

[86] Zhu M., Zhang J., Bian S., Zhang X., Shen Y., Ni Z., Xu S., Cheng C., Zheng W. (2022). Circadian Gene CSNK1D Promoted the Progression of Hepatocellular Carcinoma by Activating Wnt/β-Catenin Pathway via Stabilizing Dishevelled Segment Polarity Protein 3. Biol. Proced. Online.

[87] Lu Z., Zhou Y., Luo J., Liu Z., Xiao Z. (2026). Proposed Role of Circadian Clock Genes in Pathogenesis of HCC: Molecular Subtyping and Characterization. Biomedicines.

[88] Huang L.-H., Huang C.-Y., Liu Y.-W., Chien P.-C., Hsieh T.-M., Liu H.-T., Lin H.-P., Wu C.-J., Chuang P.-C., Hsieh C.-H. (2024). Circadian Rhythm Disruption in Hepatocellular Carcinoma Investigated by Integrated Analysis of Bulk and Single-Cell RNA Sequencing Data. Int. J. Mol. Sci..

[89] Qin H., Wang Y., Yan Y.-F., Liu J., Li Y.-Y., Xu Y.-L. (2026). Endothelial Circadian Rhythm Genes as Prognostic Modulators of Tumor Progression and Immune Interactions: Insights from Pan-Cancer Single-Cell RNA Sequencing. Int. J. Surg..

[90] Yan J., Yang X., Lu J., Wu S., Wang Y., Du Y., Zheng J., Wang F., Gao H., Yang H. (2025). Development of a Circadian-Related Prognostic Signature Highlights RBM17 as a Stemness Regulator in Liver Cancer. Cancer Cell Int..

[91] Zhang Z., Liang Z., Gao W., Yu S., Hou Z., Li K., Zeng P. (2022). Identification of Circadian Clock Genes as Regulators of Immune Infiltration in Hepatocellular Carcinoma. J. Cancer.

[92] Wang C., Zeng Q., Gül Z.M., Wang S., Pick R., Cheng P., Bill R., Wu Y., Naulaerts S., Barnoud C. (2024). Circadian Tumor Infiltration and Function of CD8+ T Cells Dictate Immunotherapy Efficacy. Cell.

[93] Fortin B.M., Pfeiffer S.M., Insua-Rodríguez J., Alshetaiwi H., Moshensky A., Song W.A., Mahieu A.L., Chun S.K., Lewis A.N., Hsu A. (2024). Circadian Control of Tumor Immunosuppression Affects Efficacy of Immune Checkpoint Blockade. Nat. Immunol..

[94] Aiello I., Fedele M.L.M., Román F., Marpegan L., Caldart C., Chiesa J.J., Golombek D.A., Finkielstein C.V., Paladino N. (2020). Circadian Disruption Promotes Tumor-Immune Microenvironment Remodeling Favoring Tumor Cell Proliferation. Sci. Adv..

[95] Filiano A.N., Millender-Swain T., Johnson R., Young M.E., Gamble K.L., Bailey S.M. (2013). Chronic Ethanol Consumption Disrupts the Core Molecular Clock and Diurnal Rhythms of Metabolic Genes in the Liver without Affecting the Suprachiasmatic Nucleus. PLoS ONE.

[96] Thomes P.G., Brandon-Warner E., Li T., Donohue T.M., Schrum L.W. (2016). Rev-Erb Agonist and TGF-β Similarly Affect Autophagy but Differentially Regulate Hepatic Stellate Cell Fibrogenic Phenotype. Int. J. Biochem. Cell Biol..

[97] Wang J., Wang Y., Lin L., Pei W., Li Y. (2025). Rev-erbα: The Circadian Guardian against NLRP3-driven Liver Fibrosis. Mol. Med. Rep..

[98] Griffett K., Bedia-Diaz G., Elgendy B., Burris T.P. (2020). REV-ERB Agonism Improves Liver Pathology in a Mouse Model of NASH. PLoS ONE.

[99] Jokl E., Llewellyn J., Simpson K., Adegboye O., Pritchett J., Zeef L., Donaldson I., Athwal V.S., Purssell H., Street O. (2023). Circadian Disruption Primes Myofibroblasts for Accelerated Activation as a Mechanism Underpinning Fibrotic Progression in Non-Alcoholic Fatty Liver Disease. Cells.

[100] Park S., Kim K., Bae I., Lee S.H., Jung J., Lee T.R., Cho E. (2018). *TIMP3* Is a CLOCK-dependent Diurnal Gene That Inhibits the Expression of UVB-induced Inflammatory Cytokines in Human Keratinocytes. FASEB J..

[101] Anea C.B., Ali M.I., Osmond J.M., Sullivan J.C., Stepp D.W., Merloiu A.M., Rudic R.D. (2010). Matrix Metalloproteinase 2 and 9 Dysfunction Underlie Vascular Stiffness in Circadian Clock Mutant Mice. Arterioscler. Thromb. Vasc. Biol..

[102] Pourcet B., Zecchin M., Ferri L., Beauchamp J., Sitaula S., Billon C., Delhaye S., Vanhoutte J., Mayeuf-Louchart A., Thorel Q. (2018). Nuclear Receptor Subfamily 1 Group D Member 1 Regulates Circadian Activity of NLRP3 Inflammasome to Reduce the Severity of Fulminant Hepatitis in Mice. Gastroenterology.

[103] Liu Z., Zhang J., Li S., Wang H., Ren B., Li J., Bao Z., Liu J., Guo M., Yang G. (2023). Circadian Control of ConA-Induced Acute Liver Injury and Inflammatory Response via Bmal1 Regulation of Junb. JHEP Rep..

[104] Li X., Wang L., Cheng X., Li M., Chen J. (2025). Circadian Disruption Aggravates Non-Alcoholic Fatty Liver Disease by Activating RIPK1-RIPK3-MLKL Axis in Mice. Sci. Rep..

[105] Yao Y., Zhang W., Ming R., Deng Q., Zuo A., Zhang S., Ying Y., Zhao Y., Ma J. (2020). Noninvasive 40-Hz Light Flicker Rescues Circadian Behavior and Abnormal Lipid Metabolism Induced by Acute Ethanol Exposure via Improving SIRT1 and the Circadian Clock in the Liver-Brain Axis. Front. Pharmacol..

[106] Li X., Zhuang R., Zhang K., Zhang Y., Lu Z., Wu F., Wu X., Li W., Zhang Z., Zhang H. (2024). Nobiletin Protects Against Alcoholic Liver Disease in Mice via the BMAL1-AKT-Lipogenesis Pathway. Mol. Nutr. Food Res..

[107] Yao Y., Zuo A., Deng Q., Liu S., Zhan T., Wang M., Xu H., Ma J., Zhao Y. (2020). Physcion Protects Against Ethanol-Induced Liver Injury by Reprogramming of Circadian Clock. Front. Pharmacol..

[108] Davis B.T., Voigt R.M., Shaikh M., Forsyth C.B., Keshavarzian A. (2017). CREB Protein Mediates Alcohol-Induced Circadian Disruption and Intestinal Permeability. Alcohol. Clin. Exp. Res..

[109] Swanson G.R., Gorenz A., Shaikh M., Desai V., Kaminsky T., Van Den Berg J., Murphy T., Raeisi S., Fogg L., Vitaterna M.H. (2016). Night Workers with Circadian Misalignment Are Susceptible to Alcohol-Induced Intestinal Hyperpermeability with Social Drinking. Am. J. Physiol. Gastrointest. Liver Physiol..

[110] Zhao K., Ni Z., Qin Y., Zhu R., Yu Z., Ma Y., Chen W., Sun Q., Wang Z., Liu Y. (2023). Disrupted Diurnal Oscillations of the Gut Microbiota in Patients with Alcohol Dependence. Front. Cell. Infect. Microbiol..

[111] Swanson G.R., Siskin J., Gorenz A., Shaikh M., Raeisi S., Fogg L., Forsyth C., Keshavarzian A. (2020). Disrupted Diurnal Oscillation of Gut-Derived Short Chain Fatty Acids in Shift Workers Drinking Alcohol: Possible Mechanism for Loss of Resiliency of Intestinal Barrier in Disrupted Circadian Host. Transl. Res..

[112] Zhang H., Zhou W., Gao P., Li Z., Li C., Li J., Bian J., Gong L., He C., Han L. (2024). Ellagic Acid Protects against Alcohol-Related Liver Disease by Modulating the Hepatic Circadian Rhythm Signaling through the Gut Microbiota–NPAS2 Axis. J. Agric. Food Chem..

[113] Gaucher J., Kinouchi K., Ceglia N., Montellier E., Peleg S., Greco C.M., Schmidt A., Forne I., Masri S., Baldi P. (2019). Distinct Metabolic Adaptation of Liver Circadian Pathways to Acute and Chronic Patterns of Alcohol Intake. Proc. Natl. Acad. Sci. USA.

[114] Shearn C.T., Anderson A.L., Devereaux M.W., Sokol R.J. (2024). Parenteral Nutrition Results in Peripheral Ileal to Hepatic Circadian Discordance in Mice. Am. J. Physiol. Gastrointest. Liver Physiol..

[115] Ceci L., Chen L., Baiocchi L., Wu N., Kennedy L., Carpino G., Kyritsi K., Zhou T., Owen T., Kundu D. (2022). Prolonged Administration of Melatonin Ameliorates Liver Phenotypes in Cholestatic Murine Model. Cell. Mol. Gastroenterol. Hepatol..

[116] Chen L., Zhou T., Wu N., O’Brien A., Venter J., Ceci L., Kyritsi K., Onori P., Gaudio E., Sybenga A. (2019). Pinealectomy or Light Exposure Exacerbates Biliary Damage and Liver Fibrosis in Cholestatic Rats through Decreased Melatonin Synthesis. Biochim. Biophys. Acta (BBA) Mol. Basis Dis..

[117] Ostrycharz E., Wasik U., Kempinska-Podhorodecka A., Banales J.M., Milkiewicz P., Milkiewicz M. (2020). Melatonin Protects Cholangiocytes from Oxidative Stress-Induced Proapoptotic and Proinflammatory Stimuli via miR-132 and miR-34. Int. J. Mol. Sci..

[118] Turco M., Cazzagon N., Franceschet I., Formentin C., Frighetto G., Giordani F., Cellini N., Mazzotta G., Costa R., Middleton B. (2018). Morning Bright Light Treatment for Sleep-Wake Disturbances in Primary Biliary Cholangitis: A Pilot Study. Front. Physiol..

[119] Curto A., Tanturli M., Iamello R.G., Rossi P., Mengozzi G., Dei L., Mello T., Innocenti T., Dragoni G., Galli A. (2026). Fatigue and Circadian Rhythm in Non-Cirrhotic Primary Biliary Cholangitis: An Exploratory Comparison with Primary Sclerosing Cholangitis and Healthy Controls. World J. Hepatol..

[120] Andrews T.S., Nakib D., Perciani C.T., Ma X.Z., Liu L., Winter E., Camat D., Chung S.W., Lumanto P., Manuel J. (2024). Single-Cell, Single-Nucleus, and Spatial Transcriptomics Characterization of the Immunological Landscape in the Healthy and PSC Human Liver. J. Hepatol..

[121] Jin C., Jiang P., Zhang Z., Han Y., Wen X., Zheng L., Kuang W., Lian J., Yu G., Qian X. (2024). Single-Cell RNA Sequencing Reveals the pro-Inflammatory Roles of Liver-Resident Th1-like Cells in Primary Biliary Cholangitis. Nat. Commun..

[122] Li X., Li Y., Xiao J., Wang H., Guo Y., Mao X., Shi P., Hou Y., Zhang X., Zhao N. (2023). Unique DUOX2+ACE2+ Small Cholangiocytes Are Pathogenic Targets for Primary Biliary Cholangitis. Nat. Commun..

[123] Braun R., Kath W.L., Iwanaszko M., Kula-Eversole E., Abbott S.M., Reid K.J., Zee P.C., Allada R. (2018). Universal Method for Robust Detection of Circadian State from Gene Expression. Proc. Natl. Acad. Sci. USA.

[124] Smith S.K., Tran P., Madden K.A., Boyd J., Braun R., Musiek E.S., Ju Y.-E.S. (2022). Validation of Blood-Based Transcriptomic Circadian Phenotyping in Older Adults. Sleep.

[125] Huang Y., Braun R. (2024). Platform-Independent Estimation of Human Physiological Time from Single Blood Samples. Proc. Natl. Acad. Sci. USA.

[126] Martens C.R., Denman B.A., Mazzo M.R., Armstrong M.L., Reisdorph N., McQueen M.B., Chonchol M., Seals D.R. (2018). Chronic Nicotinamide Riboside Supplementation Is Well-Tolerated and Elevates NAD+ in Healthy Middle-Aged and Older Adults. Nat. Commun..

[127] Dellinger R.W., Holmes H.E., Hu-Seliger T., Butt R.W., Harrison S.A., Mozaffarian D., Chen O., Guarente L. (2023). Nicotinamide Riboside and Pterostilbene Reduces Markers of Hepatic Inflammation in NAFLD: A Double-blind, Placebo-controlled Clinical Trial. Hepatology.

